# Synthesis and biological activities of petrosiols B and D†

**DOI:** 10.1039/c9ra01166h

**Published:** 2019-04-01

**Authors:** Jialin Geng, Qidong Ren, Caizhu Chang, Xinni Xie, Jun Liu, Yuguo Du

**Affiliations:** State Key Laboratory of Environmental Chemistry and Eco-Toxicology, Research Center for Eco-Environmental Sciences, Chinese Academy of Science Beijing 100085 China junliu@rcees.ac.cn; School of Chemical Sciences, University of Chinese Academy of Sciences Beijing 100049 China; School of Chemistry and Environmental Engineering, Wuhan Institute of Technology Wuhan 430205 China; National Engineering Research Center for Carbohydrate Synthesis, Jiangxi Normal University Nanchang 330022 China

## Abstract

A divergent total synthesis of natural diacetylenic tetraols, petrosiol B and petrosiol D, was accomplished by taking advantage of a carbohydrate chiral template. In particular, petrosiol B, which is the first total synthesis so far, was achieved in 13 linear steps with a 10% overall yield applying Ohira–Bestmann homologation, NaH-mediated dehydrobromination, and Cu(i)-catalyzed Cadiot–Chodkiewicz coupling as the key reaction steps. The synthetic petrosiols B and D were subjected to the study on differentiation activities toward neuronal progenitor PC12 cells. Our results suggested that both petrosiol B and petrosiol D could induce the differentiation of neuronal progenitor PC12 cells *via* the enhancement of Nrf2 activity. By comparing petrosiols B, D and their natural homologue E, petrosiol B displayed the most intensive cell differentiation activity and the highest Nrf2 activity enhancement as well.

## Abstract 2

A divergent total synthesis of natural diacetylenic tetraols, petrosiol B and petrosiol D, was accomplished by taking advantage of a carbohydrate chiral template.

## Graphical abstract

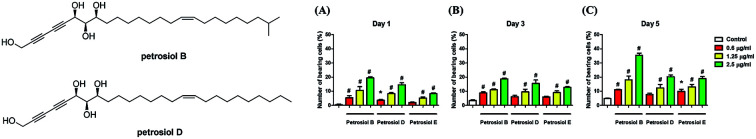

## Introduction

Polyacetylene compounds are a class of natural product with two or more conjugated acetylenic and olefinic bonds. Not surprisingly, marine organisms have become increasingly important and thoroughly investigated sources of polyacetylenes over the past few years.^1^ It is believed that these polyacetylenes are biosynthetically derived from their fatty acid progenitors *via* desaturase-promoted dehydrogenation, leading to double bonds at specific sites of the carbon chain in normally high *Z*-selectivity.^2^ Further dehydrogenation of some double bonds in the presence of acetylenases generates alkynes which play crucial roles in their biological activities.^1*a*^ Results from human nutrition research reveals that some of the polyacetylene compounds exhibit health chemopreventive effects, while some of their structural analogues are toxins with strong neurotoxicity.^3,4^ Due to their unique structural characteristics and impressive pharmacological properties such as anti-inflammatory, antimicrobial, antitumor, antiviral and neuritogenic activities, polyacetylenes have attracted significant interest in terms of the isolation, structure determination, bioactivity screening, and synthetic attempt.^1^ Recent examples of this polyacetylene family include debilisones A–F,^5^ strongylodiols A–I,^6^ ivorenolide A–B,^7^ and atractylodemaynes A–G.^8^

**Fig. 1:**
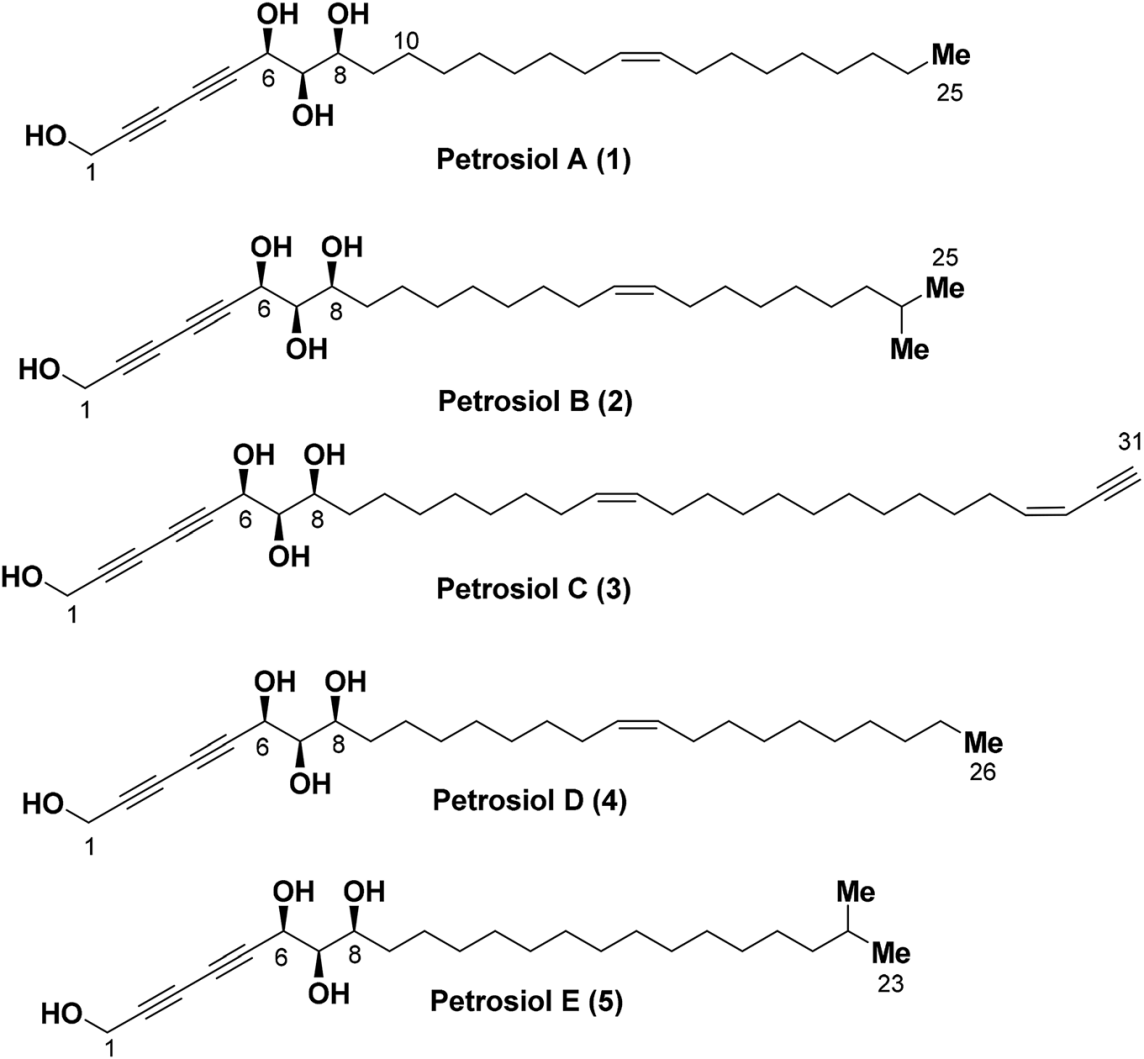
Structures of petrosiols A–E (1–5).

Petrosiols A–E (1-5) (Fig. 1) are polyacetylene-containing natural products isolated from the Okinawan marine sponge *Petrosia strongylata* by Ojika's group in 2013.^9^ The structure and absolute stereochemistry of the petrosiols was determined by derivatization of petrosiol C and a modified Mosher's method as well, sharing a common structural feature of unusual triyne–triyne inserted tetraol skeleton with different side chain residues.

Because of their novel structure feature, impressive broad-band biological activities, and most importantly the limited availability, petrosiols have attracted the attention toward the total synthesis. Srihari and co-workers reported the first total synthesis of petrosiol D using (+)-diethyl l-tartrate as starting material and Cadiot–Chodkiewicz coupling as the key step in 2013.^10^ The same group also reported the total synthesis of petrosiols A and E using a similar strategy in 2016.^11^ As part of our interests in the total synthesis of various complex natural products based on carbohydrate skeletons, we have reported the first total synthesis of petrosiol E from d-xylose in 2014.^12^ So far, there is no report regarding the syntheses of petrosiols B and C.

With the synthetic petrosiol E in hand, we studied its molecular mechanism in stimulating neuronal differentiation and antioxidative stress in PC12 neuronal progenitor cells.^13^ We found that petrosiol E could stimulate the mitogen-activated protein kinase and serine/tyrosine kinase signaling to enhance the activity of Nrf2. We also identified a dual role of petrosiol E in potentiating the differentiation of neuronal progenitors and in protecting them against arsenic-induced oxidative stress in PC12 cells. The impressive biological activity and the lack of structure–activity relationship (SAR) studies on petrosiols encouraged us to undertake a flexible and scalable synthesis of these novel natural products. Herein, we disclose the first total synthesis of petrosiol B and total synthesis of petrosiol D using readily available carbohydrate d-mannose as the chiral pool.

## Results and discussion

Based on the results from our previous biological study,^13^ petrosiols could induce nerve growth factor (NGF)-like neuronal differentiation of PC12 cells in a dose-dependent manner. It thus could provide a potential agent for the prevention and treatment of neurodegenerative diseases such as Parkinson's disease (PD) and Alzheimer disease (AD). In this regard, it is highly demanding a method to reach the common crucial tetraol skeleton of petrosiols in an efficient and easy scaling up way, although we have realized that the structure sensitivity toward oxidation and light makes them challenging synthetic target molecules.

**Scheme 1:**
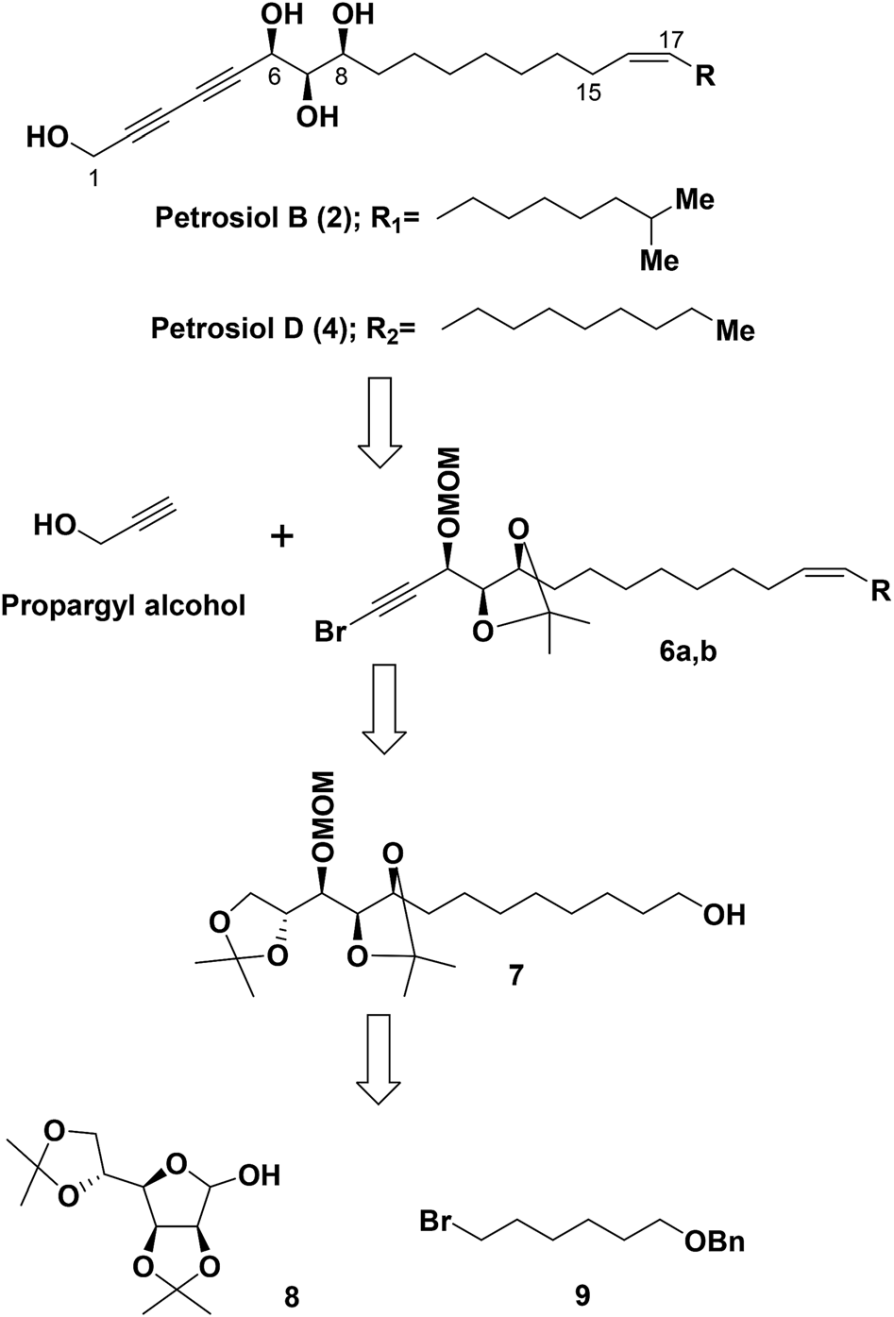
Retrosynthetic analysis of petrosiols B and D.

Our retrosynthetic analysis for the total syntheses of petrosiols B and D is presented in Scheme 1.

Accordingly, a divergent approach was designed taking advantage of the same triyne–triyne tetraol framework (C1–C17) which can be accessed by coupling of the precursor bromo-alkyne 6 with propargyl alcohol *via* Cadiot–Chodkiewicz reaction for both petrosiol B and petrosiol D. The requisite precursor 6 could be generated from the primary alcohol 7 followed by functional group transformation *via* sequential alkylation of the terminal alkyne, Lindlar catalyst promoted *cis*-reduction, acetonide cleavage, and Corey–Fuchs reaction. As for the key intermediate 7, it could be obtained by coupling of bromide 9 and hemiacetal 8*via* a multi-step sequential modification. Details of the studies thus undertaken are described below.

**Scheme 2:**
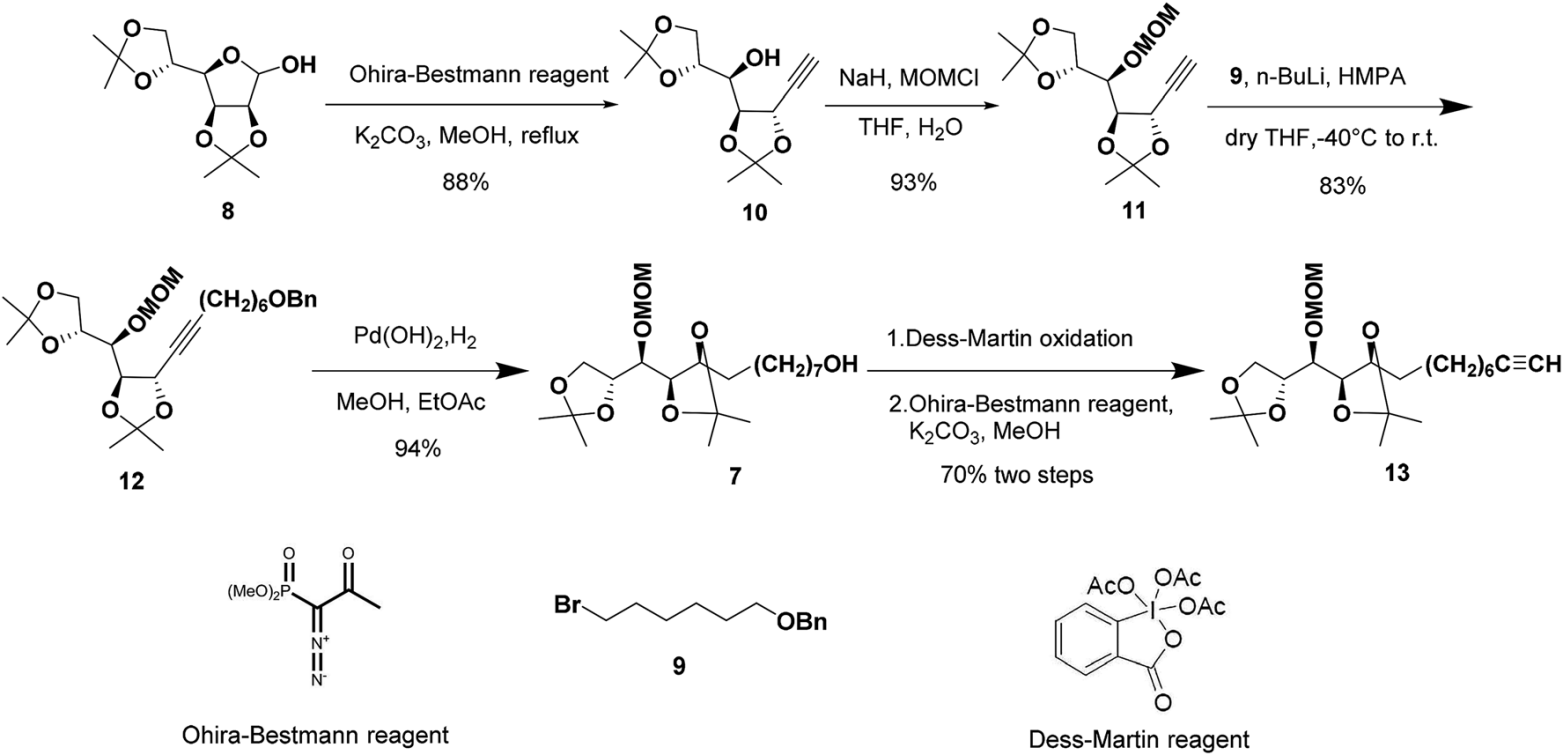
Synthesis of the C5–C16 fragment.

The synthetic sequence commenced with the formation of the terminal alkyne 10 from commercially available 2,3:5,6-di-*O*-isopropylidene-α-d-mannofuranose (8) as the starting material following a modified procedure from Lievre's report (Scheme 2).^14^ We thought that a total synthesis of the target petrosiols from a chiral carbohydrate material with a reliably assigned absolute configuration might substantiate the reported structural assignment. Thus, the masked carbonyl group of hemiacetal 8 was converted into the corresponding terminal alkyne by addition of Ohira–Bestmann reagent and K_2_CO_3_ in refluxing methanol.^15,16^ The desired epimerization at the propargylic stereocenter (C-5) occurred smoothly and confirmed by an up-field shifting of H5 signal (4.66 ppm, *J* = 7.6, 2.0 Hz).^14^ The full inversion of stereocenter could be attributed to the greater thermodynamic stability of the *trans* over the *cis* C4–C5 acetonide structure.^16^

To differentiate the secondary alcohol of 10 from its latent primary alkyl hydroxyl group introduced later, alkyne 10 was exposed to excess MOMCl in dichloromethane at rt for 48 h with DIPEA as the base. However, the MOM-protected product 11 was isolated in only 10% yield together with 71% of recovered 10. Attempts to improve the yield of this protection by changing solvents or elevating reaction temperature were fruitless (Table 1, entries 1–4). Other attempts to protect the secondary alcohol using TBDPS as protection group also failed (entries 5–6). Using stronger base such as DMAP or NaH did not improve the reaction yield at all. To our surprise, the desired MOM-protected product 11 was eventually obtained in 93% yield in the presence of NaH in THF containing two drops of water at room temperature (entry 8). After reviewing the literature, we recognize that catalytic amounts of water and NaH produce highly active NaOH, which is the real base to promote the reaction.^17^ We assumed that the exothermic process when adding water into the reaction mixture also facilitated the reaction in some extent.^18^

**Table 1:** Optimization of the reaction conditions

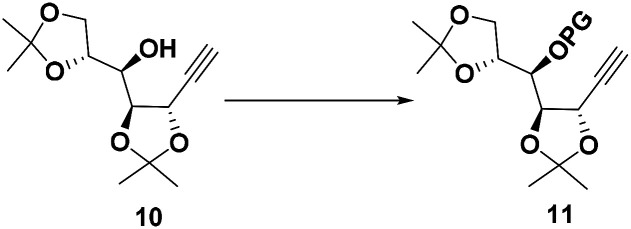					
Entry	Conditions	Solvent	*T* (°C)	Time (h)	Yield (%)
1	MOMCl (2 eq.), DIPEA (3 eq.)	DCM	20	48	<10
2	MOMCl (5 eq.), DIPEA (3 eq.)	DCM	Reflux	48	18
3	MOMCl (2 eq.), DIPEA (3 eq.), DMAP (cat)	THF	20	48	<10
4	MOMCl (2 eq.), DIPEA (3 eq.), DMAP (cat)	DCM	Reflux	48	20
5	TBDPSCl (1.5 eq.), imidazole (4 eq.)	DMF	20	48	<5
6	TBDPSCl (3 eq.), imidazole (4 eq.)	DMF	80	48	<5
7	MOMCl (3 eq.), NaH (5 eq.)	THF	20	7	<5
8	MOMCl (3 eq.), NaH (5 eq.), with few drops of water	THF	20	5	93

Terminal alkyne 11 was then alkylated with bromide 9 in the presence of HMPA leading to the desired alkyne 12 in a yield of 83%. Hydrogenation of 12 in methanol/ethyl acetate removed benzyl group and saturated the triple bond at the same time gave 7 in excellent yield. The primary alcohol 7 was oxidized to the corresponding aldehyde by Dess–Martin oxidation^19^ and the crude aldehyde was used directly for the Ohira–Bestmann homologation^20^ afforded 13 in 70% yield over two steps.

**Scheme 3:**
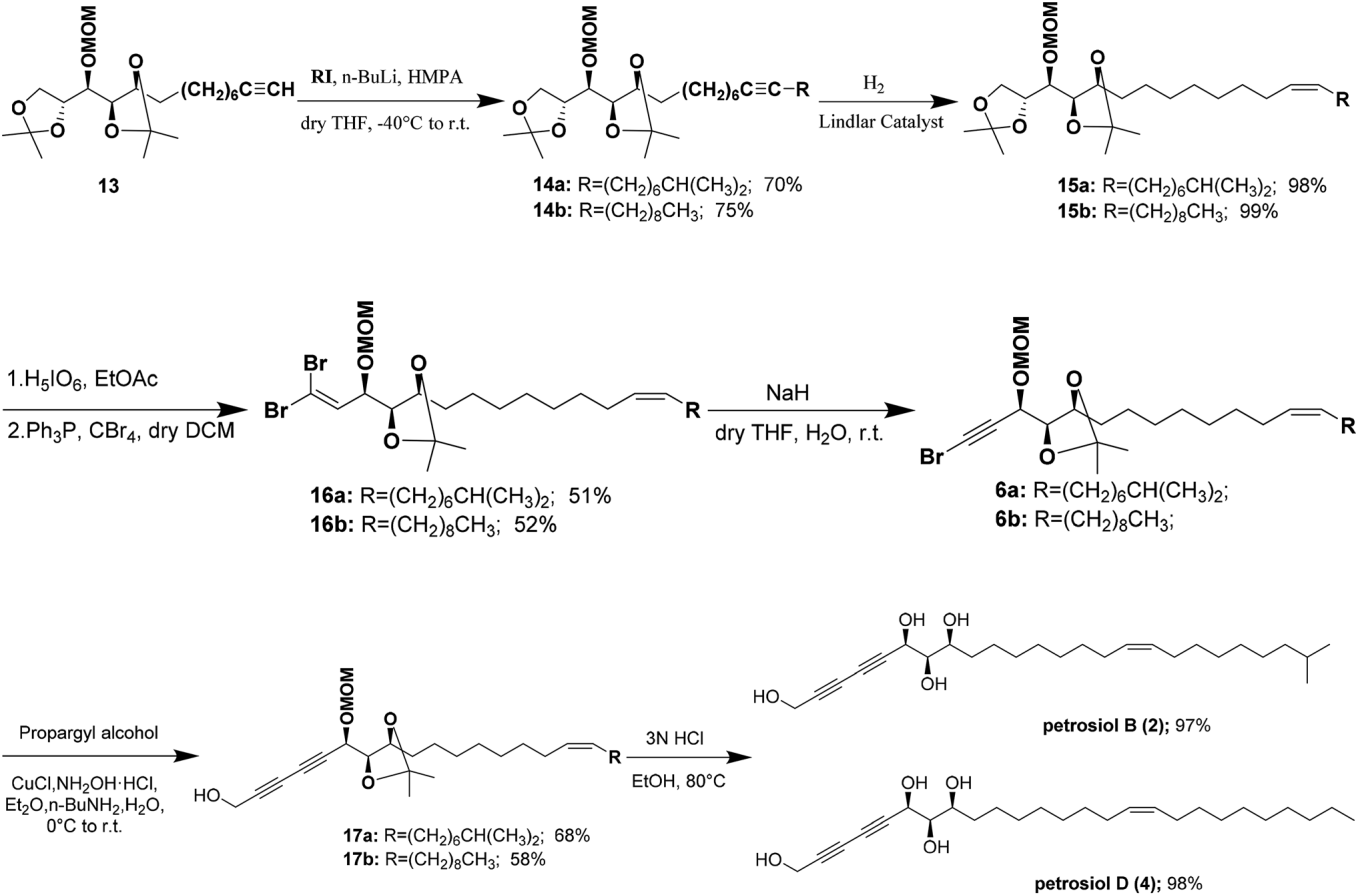
Total synthesis of petrosiols B and D.

Alkylation of the terminal alkyne 13 with iodoalkanes afforded compounds 14a,b, corresponding to petrosiols B and D respectively, in good yields (Scheme 3). Partial hydrogenation of 14a,b with Lindlar catalyst (5% Pd on CaCO_3_ poisoned with lead) under H_2_ atmosphere afforded the *cis*-olefins 15a,b with excellent *Z*/*E* selectivity (>20 : 1). Regioselective oxidative cleavage of the terminal isopropylidene group in 15a,b was performed with periodic acid in dry ethyl acetate affording one-carbon diminished aldehyde,^20^ which was immediately treated with CBr_4_/PPh_3_ in DCM^21,22^ to generate 1,l-dibromoalkenes 16a,b in about 51–52% yield. NaH-mediated dehydrobromination^12,23^ of dibromoalkenes in wet THF at room temperature provided monobromoalkynes 6a,b in excellent yield. Cu(i)-catalyzed Cadiot–Chodkiewicz cross coupling of propargyl alcohol with monobromoalkynes 6a,b afforded the corresponding diynes 17a,b.^24^ Finally, one-pot global deprotection of 17a,b were achieved with 3 N HCl in refluxing ethanol, delivering petrosiol B (2) in 97% yield and petrosiol D (4) in 98% yield, respectively. The spectroscopic data (^1^H, ^13^C NMR, and HRMS) and specific rotation data of both synthetic petrosiol B and petrosiol D were in good agreement with those of the natural products (see the ESI†).

## Bioactivity section

**Fig. 2:**
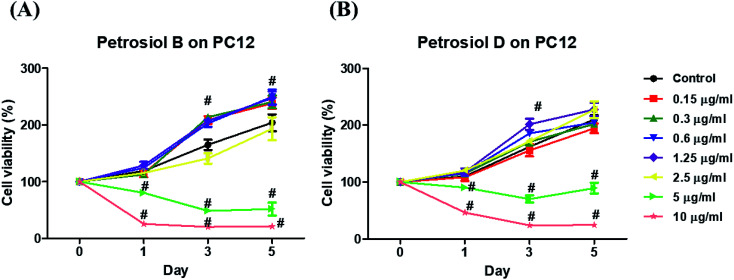
Screening of sub-lethal concentrations of petrosiols B (A) and D (B) on PC12 cells. Cell viability was determined through CCK-8 assay at different concentrations of petrosiols B and D for 1, 3, and 5 d. ^#^*P* < 0.001, relative to untreated control.

Biological activity of the synthetic petrosiols B and D were investigated following our previous evidences on homologue E induced PC12 cells differentiation.^13^ To find out the feasible concentrations in differentiation experiment, petrosiols ranging from 0.15 μg mL^−1^ to 10 μg mL^−1^ were subjected to PC12 cell to evaluate the cell viability using the Cell Counting Kit-8 (CCK-8). As shown in Fig. 2, petrosiol displayed no obvious effects on cell viability from day 1 to day 5 when the cell was treated with petrosiol (B/D) below the concentration of 5 μg mL^−1^. In particular, petrosiol B inhibited PC12 cell viability by about 74% (*P* < 0.001) after 1 day treatment and further repressed by about 80% (*P* < 0.001) after 5 day treatment at 10 μg mL^−1^ (Fig. 2A), while petrosiol D inhibited cell viability by about 54% (*P* < 0.001) after 1 day and about 75% (*P* < 0.001) after 5 day at 10 μg mL^−1^ (Fig. 2B). Therefore, concentrations lower than 5 μg mL^−1^ were chosen for the synthetic petrosiols in the following biological experiments.

**Fig. 3:**
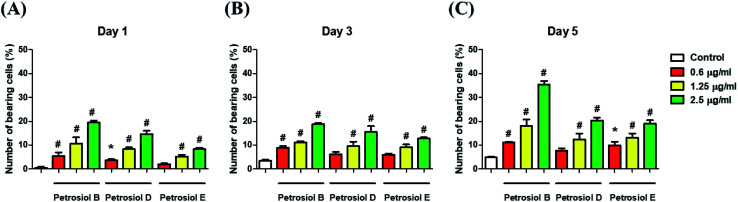
Petrosiols B and D promoted neurite outgrowth and activity of Nrf2 in PC12 cells. Petrosiol B, D and E promoted neurite outgrowth in PC12 cells on day 1 (A), 3 (B), and 5 (C). *P < 0.05, relative to control. ^#^*P* < 0.001, relative to control.

**Fig. 4:**
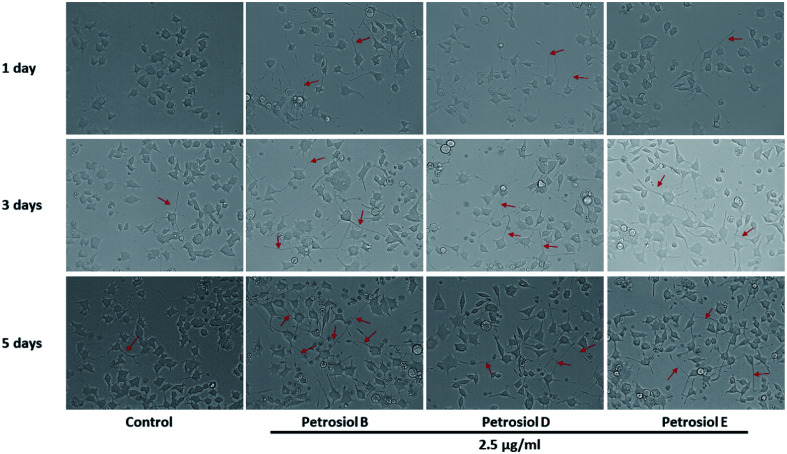
The morphology changes of PC12 cells after petrosiols B, D, and E induction at 2.5 μg mL^−1^ at day 3 and day 5.

To evaluate the effect of petrosiol on neuronal progenitors differentiation, PC12 cells were treated with petrosiols at different concentrations and under varying exposure time. As shown in Fig. 3 and 4, treatment of PC12 cells with petrosiols B, D and E all significantly increased the number of neurites in cells in a dose- and time-dependent manner compared to control treatment, and petrosiol B showed strongest activity on neuronal differentiation. Specifically on day 5 at 2.5 μg mL^−1^, petrosiol B induced about 35% (*P* < 0.001) of cells generating neurite outgrowth, while 20% (*P* < 0.001) petrosiol D, 19% (*P* < 0.001) for petrosiol E under the same incubation conditions (Fig. 3C). Interestingly, petrosiols D and E induced a comparative differentiating cells on day 5 (Fig. 3C) though D showed stronger inducing effect than E on day 1 and 3 (Fig. 3A and B). The current result suggested that petrosiol compounds triggered neuronal differentiation of PC12 cells, though petrosiol B exhibited stronger effect than its homologues D and E.

**Fig. 5:**
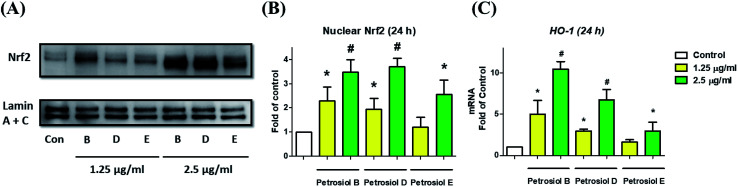
Petrosiols upregulated Nrf2 protein level in nuclear. (A) Protein levels in PC12 cell nuclear were determined *via* western blot. (B) Quantitative analysis of Nrf2 protein abundance was conducted with ImageJ software. Lamin A + C served as loading control. (C) The mRNA levels of HO-1 expression upon various concentrations of petrosiols B, D and E for 24 h. *P < 0.05, relative to control. ^#^*P* < 0.001, relative to control.

We also investigated the molecular mechanism concealing the petrosiol-induced neuronal differentiation. Our previous study indicated that petrosiol E induced PC12 differentiation by enhancing the activity of Nrf2 which is indispensable in an augmented differentiation process. We thus explored Nrf2 activity of PC12 cells in the presence of petrosiols B and D by detecting the contents of Nrf2 protein in nucleus, respectively. As shown in Fig. 5, petrosiols (at 1.25 and 2.5 μg mL^−1^) significantly upregulated the contents of Nrf2 in nuclear portion after treatment for 24 h, relative to untreated control cells. After 24 h treatment with petrosiols B, D and E at 2.5 μg mL^−1^, the nuclear Nrf2 level was increased by 3.5- (*P* < 0.001), 3.7- (*P* < 0.001) and 2.6-fold (*P* < 0.05) compared to the untreated control (Fig. 5B), respectively. To further confirm the petrosiols induced Nrf2 activation, downstream target gene heme oxygenase-1 (HO-1)^25^ of Nrf2 was also checked at mRNA level upon petrosiol exposure. It was found that petrosiols B, D and E at 2.5 μg mL^−1^ increased mRNA level of HO-1 by 10.4- (*P* < 0.001), 6.7- (*P* < 0.001) and 2.9-fold (*P* < 0.05) relative to the untreated control, respectively, which were consistent with the enhancement of nuclear Nrf2 protein (Fig. 5C). These findings strongly indicated that petrosiols could enhance neuronal differentiation through activating Nrf2.

## Conclusions

In conclusion, we have achieved the first total synthesis of petrosiol B and a total synthesis of petrosiol D at the same time using readily available carbohydrate d-mannose as the chiral template. The synthetic petrosiols (B, D and E) were found to elicit the differentiation of neuronal progenitor PC12 cells through an enhanced Nrf2 activity in cell nuclear. Among them, petrosiol B presented the best potential in this regards. Further efforts for the divergent syntheses and SAR studies of other petrosiol-derived polyacetylenes^1,26^ are currently underway in our laboratory.

## Experimental

### General methods

Unless noted otherwise, commercially available materials were used without further purification. Yields refer to chromatographically and spectroscopically (^1^H NMR) homogeneous materials. All solvents were dried according to the established procedures ahead of use. Flash chromatography (FC) was performed using silica gel (200–300 meshes) according to the standard protocol. All reactions under standard conditions were monitored by thin-layer chromatography (TLC) on gel F254 plates. Optical rotations were measured using a polarimeter with a thermally jacketed 2.5 cm cell at approximately 25 °C. High-resolution mass spectrometry data (HRMS) were acquired using a Q-TOF analyzer in methanol as solvent. ^1^H NMR, ^13^C NMR were measured on 400 MHz or 100 MHz spectrometers. Chemicals shifts (*δ*) were expressed in ppm relative to residual CHCl_3_, CD_3_OD or TMS. Multiplicity is tabulated as s for singlet, d for doublet, t for triplet, q for quadruplet, and m for multiplet and br when the signal in question is broadened.

### Synthesis of compound 10

MeOH (10 mL) was added to a mixture of 8 (3.0 g, 11.5 mmol) and K_2_CO_3_ (4.0 g, 28.9 mmol). The mixture was stirred under an argon atmosphere and allowed to reflux. Dimethyl(1-diazo-2-oxopropyl)phosphonate (4.33 mL, 28.8 mmol) was added dropwise within 6 h, at the end of which time the mixture was cooled to room temperature, then it was filtered on glass-frit and concentrated. After an extraction with EtOAc/H_2_O, the combined organic extracts was dried over Na_2_SO_4_ and concentrated. The crude residue was purified by flash chromatography (petroleum ether/EtOAc 4 : 1) to afford 10 as a syrup. Yield: 2.6 g (88%): [*α*]^28^_D_ = −25.7 (*c* 1.4, CHCl_3_); ^1^H NMR (400 MHz, CDCl_3_) *δ* 4.66 (dd, *J* = 7.6, 2.0 Hz, 1H), 4.28 (d, *J* = 7.6 Hz, 1H), 4.13–3.98 (m, 3H), 3.56 (dd, *J* = 7.4, 2.3 Hz, 1H), 2.54 (d, *J* = 2.1 Hz, 1H), 1.51 (s, 3H), 1.43 (d, *J* = 2.5 Hz, 6H), 1.36 (s, 3H); ^13^C NMR (100 MHz, CDCl_3_) *δ* 111.2, 109.7, 81.0, 80.7, 76.3, 75.1, 70.0, 67.0, 66.8, 27.0, 26.8, 26.3, 25.5; HRMS (ESI): calcd. for C_13_H_20_O_5_K^+^ [M + K]^+^, 295.0942; found 295.0983. The ^1^H and ^13^C NMR spectra were identical to those reported in ref. 14.

### Synthesis of compound 11

To a stirred solution of 10 (889 mg, 3.47 mmol) in dry THF (50 mL) were added NaH (694 mg, 17.4 mmol) and methoxymethyl chloride (0.79 mL, 10.4 mmol). After 5 min, deionized water (50 μL) was added slowly into the mixture *via* syringe at room temperature and it will be exothermic. The reaction mixture was stirred for 5 h and quenched with saturated NH_4_Cl aqueous solution. The organic phase in the resulting solution mixture was extracted with EtOAc (3 times, 50 mL each), dried over Na_2_SO_4_, filtered, and finally evaporated under a reduced pressure. The residue was purified by chromatography on silica gel (petroleum ether/EtOAc 7 : 1) to afford 11 as a colorless oil. Yield: 967 mg (93%): [*α*]^25^_D_ = −25.0 (*c* 2.1, CHCl_3_); ^1^H NMR (400 MHz, CDCl_3_) *δ* 4.83 (d, *J* = 6.8 Hz, 1H), 4.74 (d, *J* = 6.7 Hz, 1H), 4.65 (dd, *J* = 7.5, 2.0 Hz, 1H), 4.24–4.17 (m, 1H), 4.15 (dd, *J* = 7.5, 3.7 Hz, 1H), 4.09–3.99 (m, 2H), 3.85 (t, *J* = 4.3 Hz, 1H), 3.42 (s, 3H), 2.54 (d, *J* = 2.0 Hz, 1H), 1.48 (s, 3H), 1.42 (d, *J* = 3.0 Hz, 6H), 1.36 (s, 3H); ^13^C NMR (100 MHz, CDCl_3_) *δ* 110.9, 109.0, 98.3, 82.2, 81.0, 76.7, 75.8, 74.9, 66.8, 65.8, 56.4, 26.7, 26.6, 26.4, 25.5; HRMS (ESI): calcd. for C_15_H_24_O_6_Na^+^ [M + Na]^+^, 323.1465; found 323.1457.

### Synthesis of compound 12

*n*-Butyllithium (2.5 M in hexanes, 1.33 mL, 3.32 mmol) was added dropwise to a stirred solution of terminal alkyne 11 (830 mg, 2.77 mmol) and HMPA (0.73 mL, 4.15 mmol) in dry THF (30 mL) at −40 °C. The reaction mixture was stirred at −40 °C for 20 minutes, then added 9 gave an orange colored solution. The reaction mixture was stirred at room temperature overnight and quenched with saturated NH_4_Cl aqueous solution. The organic phase in the resulting solution mixture was extracted with EtOAc (3 times, 50 mL each), dried over Na_2_SO_4_, filtered, and finally evaporated under a reduced pressure. The residue was purified by chromatography on silica gel (petroleum ether/EtOAc 7 : 1) to afford 12 as a colorless oil. Yield: 967 mg (83%): [*α*]^25^_D_ = −21.6 (*c* 0.4, CHCl_3_); ^1^H NMR (400 MHz, CDCl_3_) *δ* 7.36–7.26 (m, 5H), 4.84 (d, *J* = 6.6 Hz, 1H), 4.73 (d, *J* = 6.6 Hz, 1H), 4.60 (dt, *J* = 7.7, 1.9 Hz, 1H), 4.49 (s, 2H), 4.24–4.16 (m, 1H), 4.06–3.97 (m, 3H), 3.86 (t, *J* = 4.0 Hz, 1H), 3.45 (t, *J* = 6.6 Hz, 2H), 3.41 (s, 3H), 2.21 (td, *J* = 7.1, 1.9 Hz, 2H), 1.65–1.57 (m, 2H), 1.56–1.44 (m, 5H), 1.43–1.34 (m, 13H); ^13^C NMR (100 MHz, CDCl_3_) *δ* 138.8, 128.5, 127.8, 127.7, 110.2, 108.9, 98.2, 87.9, 82.2, 76.8, 76.6, 75.3, 73.1, 70.5, 67.4, 65.6, 56.4, 29.8, 28.9, 28.6, 26.8, 26.7, 26.6, 25.9, 25.5, 18.9; HRMS (ESI): calcd. for C_28_H_43_O_7_^+^[M + H]^+^, 491.3003, found 491.3004.

### Synthesis of compound 7

To a solution of 12 (900 mg, 1.83 mmol) in 30 mL of methanol and ethyl acetate (v/v = 1 : 2) was added palladium hydroxide (moisture *ca.* 60%, 20% Pd, 51 mg) at room temperature under H_2_ atmosphere. The mixture was stirred at room temperature for 1.5 h, filtered through a plug of silica gel, washed with ethyl acetate, and concentrated to afford crude compound 7 as a colorless oil which was used without further purification. Yield: 697 mg (94%): [*α*]^25^_D_ = −30.4 (*c* 0.4, CHCl_3_); ^1^H NMR (400 MHz, CDCl_3_) *δ* 4.83 (d, *J* = 6.7 Hz, 1H), 4.69 (d, *J* = 6.7 Hz, 1H), 4.20–4.13 (m, 1H), 4.06–3.98 (m, 3H), 3.73–3.69 (m, 1H), 3.66 (dd, *J* = 8.1, 2.8 Hz, 1H), 3.60 (t, *J* = 6.6 Hz, 2H), 3.39 (s, 3H), 1.59–1.42 (m, 5H), 1.40–1.25 (m, 21H); ^13^C NMR (100 MHz, CDCl_3_) *δ* 108.7, 108.6, 98.4, 81.5, 77.3, 76.8, 76.1, 65.9, 63.1, 56.4, 33.2, 32.9, 29.8, 29.6, 29.5, 27.6, 26.8, 26.6, 26.3, 25.9, 25.4; HRMS (ESI): calcd. for C_21_H_40_O_7_Na^+^ [M + Na]^+^, 427.2666, found 427.2656.

### Synthesis of compound 13

To a solution of 7 (160 mg, 0.40 mmol) in 10 mL of DCM was added NaHCO_3_ (66 mg, 0.80 mmol) and Dess–Martin periodinane (251 mg, 0.40 mmol) at 0 °C. The mixture was stirred at room temperature for 0.5 h and quenched with saturated NaHCO_3_ aqueous solution. The organic phase in the resulting solution mixture was extracted with DCM (3 times, 50 mL each), dried over Na_2_SO_4_, filtered, and finally evaporated under a reduced pressure. The colorless oil was extracted with petroleum ether and DCM, concentrate the supernatant to afford crude aldehyde which was used without further purification.

Dimethyl-2-oxopropylphosphonate (152 mg, 119 μL, 0.80 mmol) was added to a suspension of K_2_CO_3_ (164 mg, 1.20 mmol) and the crude aldehyde in MeOH (10 mL) under N_2_ atmosphere. The mixture was stirred at room temperature for 4.5 h. The blue mixture was concentrated, extracted with EtOAc, dried over Na_2_SO_4_, filtered, and finally evaporated under a reduced pressure. The residue was purified by chromatography on silica gel (petroleum ether/EtOAc 7 : 1) to afford 13 as a colorless oil. Yield: 119 mg (66% for three steps): [*α*]^25^_D_ = −26.4 (*c* 2.0, CHCl_3_); ^1^H NMR (400 MHz, CDCl_3_) *δ* 4.85 (d, *J* = 6.7 Hz, 1H), 4.70 (d, *J* = 6.8 Hz, 1H), 4.24–4.13 (m, 1H), 4.08–3.97 (m, 3H), 3.76–3.70 (m, 1H), 3.68 (dd, *J* = 8.0, 2.8 Hz, 1H), 3.40 (s, 3H), 2.17 (td, *J* = 7.0, 2.6 Hz, 2H), 1.93 (t, *J* = 2.6 Hz, 1H), 1.63–1.45 (m, 5H), 1.43–1.24 (m, 19H); ^13^C NMR (100 MHz, CDCl_3_) *δ* 108.69, 108.66, 98.4, 84.9, 81.6, 77.3, 76.8, 76.1, 68.3, 66.0, 56.5, 33.3, 29.7, 29.2, 28.8, 28.6, 27.7, 26.8, 26.7, 26.3, 25.5, 18.6; HRMS (ESI): calcd. for C_22_H_38_O_6_Na^+^ [M + Na]^+^, 421.2561, found 421.2569.

### Synthesis of compound 14a

*n*-Butyllithium (2.5 M in hexanes, 0.14 mL, 0.36 mmol) was added dropwise to a stirred solution of terminal alkyne 13 (110 mg, 0.28 mmol) and HMPA (72 μL, 0.41 mmol) in dry THF (10 mL) at −40 °C. The reaction mixture was stirred at room temperature and added 1-iodo-7-methyloctane gave an orange colored solution. Quenched with saturated NH_4_Cl aqueous solution after 4.5 h. The organic phase in the resulting solution mixture was extracted with EtOAc (3 times, 50 mL each), dried over Na_2_SO_4_, filtered, and finally evaporated under a reduced pressure. The residue was purified by chromatography on silica gel (petroleum ether/EtOAc 12 : 1) to afford 14a as a colorless oil. Yield: 101 mg (70%): [*α*]^25^_D_ = −20.5 (*c* 0.8, CHCl_3_); ^1^H NMR (400 MHz, CDCl_3_) *δ* 4.84 (d, *J* = 6.6 Hz, 1H), 4.70 (d, *J* = 6.6 Hz, 1H), 4.23–4.12 (m, 1H), 4.07–3.98 (m, 3H), 3.75–3.70 (m, 1H), 3.67 (dd, *J* = 8.0, 2.8 Hz, 1H), 3.40 (s, 3H), 2.18–2.07 (m, 4H), 1.60–1.42 (m, 8H), 1.41–1.29 (m, 21H), 1.28–1.23 (m, 4H), 1.18–1.09 (m, 2H), 0.85 (d, *J* = 6.6 Hz, 6H); ^13^C NMR (100 MHz, CDCl_3_) *δ* 108.68, 108.65, 98.4, 81.6, 80.5, 80.3, 77.4, 76.8, 76.1, 66.0, 56.4, 39.2, 33.3, 29.8, 29.6, 29.4, 29.3, 29.3, 29.1, 29.0, 28.1, 27.7, 27.5, 26.8, 26.7, 26.4, 25.5, 22.8, 19.0, 18.9; HRMS (ESI): calcd. for C_31_H_56_O_6_Na^+^ [M + Na]^+^, 547.3969, found 547.3969.

### Synthesis of compound 14b

*n*-Butyllithium (2.5 M in hexanes, 91 μL, 0.23 mmol) was added dropwise to a stirred solution of terminal alkyne 13 (70 mg, 0.18 mmol) and HMPA (46 μL, 0.26 mmol) in dry THF (8 mL) at −40 °C. The reaction mixture was stirred at room temperature and added 1-iodononane gave an orange colored solution. Quenched with saturated NH_4_Cl aqueous solution after 20 h. The organic phase in the resulting solution mixture was extracted with EtOAc (3 times, 30 mL each), dried over Na_2_SO_4_, filtered, and finally evaporated under a reduced pressure. The residue was purified by chromatography on silica gel (petroleum ether/EtOAc 7 : 1) to afford 14b as a colorless oil. Yield: 67 mg (75%): [*α*]^25^_D_ = −18.4 (*c* 1.4, CHCl_3_); ^1^H NMR (400 MHz, CDCl_3_) *δ* 4.85 (d, *J* = 6.7 Hz, 1H), 4.71 (d, *J* = 6.7 Hz, 1H), 4.22–4.13 (m, 1H), 4.11–3.91 (m, 3H), 3.77–3.71 (m, 1H), 3.68 (dd, *J* = 8.0, 2.8 Hz, 1H), 3.41 (s, 3H), 2.16–2.09 (m, 4H), 1.62–1.42 (m, 9H), 1.41–1.12 (m, 29H), 0.87 (t, *J* = 6.6 Hz, 3H); ^13^C NMR (100 MHz, CDCl_3_) *δ* 108.70, 108.66, 98.4, 81.6, 80.5, 80.3, 77.4, 76.9, 76.1, 66.0, 56.5, 33.3, 32.1, 29.8, 29.7, 29.5, 29.4, 29.3, 29.1, 29.0, 27.7, 26.9, 26.7, 26.4, 25.5, 22.9, 19.0, 14.3; HRMS (ESI) calcd for C_31_H_56_O_6_Na^+^ [M + Na]^+^ 547.3969, found 547.3975.

### Synthesis of compound 15a

To a solution of 14a (40 mg, 0.076 mmol) in 6 mL of methanol and ethyl acetate was added Lindlar catalyst (12 mg, 0.023 mmol) at room temperature under H_2_ atmosphere. The mixture was stirred at room temperature for 1.5 h, filtered through a plug of silica gel, washed with ethyl acetate, and concentrated to afford 15a as a colorless oil which was used without further purification. Yield: 39 mg (98%): [*α*]^25^_D_ = −15.0 (*c* 0.8, CHCl_3_); ^1^H NMR (400 MHz, CDCl_3_) *δ* 5.43–5.25 (m, 2H), 4.84 (d, *J* = 6.8 Hz, 1H), 4.71 (d, *J* = 6.8 Hz, 1H), 4.25–4.13 (m, 1H), 4.09–3.97 (m, 3H), 3.75–3.71 (m, 1H), 3.68 (dd, *J* = 8.0, 2.9 Hz, 1H), 3.40 (s, 3H), 2.07–1.92 (m, 4H), 1.61–1.45 (m, 4H), 1.43–1.22 (m, 29H), 1.20–1.09 (m, 2H), 0.85 (d, *J* = 6.7 Hz, 6H); ^13^C NMR (100 MHz, CDCl_3_) *δ* 130.1, 130.0, 108.7, 108.6, 98.4, 81.6, 77.4, 76.1, 66.0, 56.4, 39.2, 33.3, 30.0, 29.95, 29.94, 29.90, 29.6, 29.5, 29.4, 28.2, 27.7, 27.6, 27.40, 27.39, 26.8, 26.7, 26.4, 25.5, 22.8; HRMS (ESI) calcd for C_31_H_58_O_6_Na^+^ [M + Na]^+^ 549.4126, found 549.4125.

### Synthesis of compound 15b

To a solution of 14b (300 mg, 0.59 mmol) in 40 mL of methanol and ethyl acetate was added Lindlar catalyst (30 mg, 0.059 mmol) at room temperature under H_2_ atmosphere. The mixture was stirred at room temperature for 1.5 h, filtered through a plug of silica gel, washed with ethyl acetate, and concentrated to afford 15b as a colorless oil which was used without further purification. Yield: 298 mg (99%): [*α*]^25^_D_ = −23.9 (*c* 0.6, CHCl_3_); ^1^H NMR (400 MHz, CDCl_3_) *δ* 5.41–5.28 (m, 2H), 4.85 (d, *J* = 6.8 Hz, 1H), 4.71 (d, *J* = 6.8 Hz, 1H), 4.24–4.14 (m, 1H), 4.09–3.96 (m, 3H), 3.76–3.71 (m, 1H), 3.68 (dd, *J* = 8.0, 2.8 Hz, 1H), 3.40 (s, 3H), 2.09–1.88 (m, 4H), 1.64–1.44 (m, 3H), 1.43–1.20 (m, 35H), 0.87 (t, *J* = 6.7 Hz, 3H); ^13^C NMR (100 MHz, CDCl_3_) *δ* 130.1, 130.0, 108.69, 108.65, 98.4, 81.6, 77.4, 76.1, 66.0, 56.5, 33.3, 32.1, 29.96, 29.95, 29.9, 29.80, 29.76, 29.7, 29.53, 29.51, 29.4, 27.7, 27.41, 27.39, 26.8, 26.7, 26.4, 25.5, 22.9, 14.3; HRMS (ESI) calcd for C_31_H_58_O_6_Na^+^ [M + Na]^+^ 549.4126, found 549.4128.

### Synthesis of compound 16a

A solution of compound 15a (32 mg, 0.061 mmol) and H_5_IO_6_ (28 mg, 0.12 mmol) in 2 mL EtOAc was stirred for 1 h. The reaction mixture was filtered, and the filtrate was evaporated to afford aldehyde as an orange-red oil.

To a solution of triphenylphosphine (127 mg, 0.49 mmol) in 2 mL dry DCM was added carbon tetrabromide (81 mg, 0.24 mmol) at 0 °C. The resulting mixture was stirred for 5 min, and then aldehyde was added slowly *via* syringe. Quenched with saturated NaHCO_3_ aqueous solution after 2 hours. The organic phase in the resulting solution mixture was extracted with DCM (3 times, 20 mL each), dried over Na_2_SO_4_, filtered, and finally evaporated under a reduced pressure. The residue was purified by chromatography on silica gel (petroleum ether/EtOAc 20 : 1) to afford 16a as a colorless oil. Yield: 19 mg (51% for three steps): [*α*]^25^_D_ = −42.7 (*c* 0.6, CHCl_3_); ^1^H NMR (400 MHz, CDCl_3_) *δ* 6.44 (d, *J* = 9.1 Hz, 1H), 5.42–5.28 (m, 2H), 4.71 (d, *J* = 6.8 Hz, 1H), 4.61 (d, *J* = 6.8 Hz, 1H), 4.40 (dd, *J* = 9.1, 5.3 Hz, 1H), 4.03–3.95 (m, 1H), 3.73 (dd, *J* = 7.8, 5.4 Hz, 1H), 3.40 (s, 3H), 2.12–1.89 (m, 4H), 1.61–1.45 (m, 4H), 1.42 (d, *J* = 1.6 Hz, 6H), 1.38–1.21 (m, 17H), 1.18–1.10 (m, 2H), 0.86 (d, *J* = 6.6 Hz, 6H); ^13^C NMR (100 MHz, CDCl_3_) *δ* 135.8, 130.2, 130.0, 109.4, 94.7, 93.8, 82.1, 77.1, 76.3, 56.0, 39.3, 33.9, 30.0, 29.97, 29.96, 29.7, 29.59, 29.56, 29.4, 28.2, 27.7, 27.6, 27.43, 27.42, 26.9, 26.2, 22.9; HRMS (ESI) calcd for C_28_H_50_Br_2_O_4_K^+^ [M + K]^+^ 647.1707, found 647.1713.

### Synthesis of compound 16b

A solution of compound 15b (262 mg, 0.51 mmol) and H_5_IO_6_ (234 mg, 1.02 mmol) in 20 mL EtOAc was stirred for 1 h. The reaction mixture was filtered, and the filtrate was evaporated to afford aldehyde as a orange red oil.

To a solution of triphenylphosphine (1.08 g, 4.12 mmol) in 20 mL dry DCM was added carbon tetrabromide (680 mg, 2.06 mmol) at 0 °C. The resulting mixture was stirred for 5 min, and then aldehyde was added slowly *via* syringe. Quenched with saturated NaHCO_3_ aqueous solution after 2 hours. The organic phase in the resulting solution mixture was extracted with DCM (3 times, 100 mL each), dried over Na_2_SO_4_, filtered, and finally evaporated under a reduced pressure. The residue was purified by chromatography on silica gel (petroleum ether/EtOAc 20 : 1) to afford 16b as a colorless oil. Yield: 166 mg (52% for three steps): [*α*]^25^_D_ = −64.9 (*c* 0.2, CHCl_3_); ^1^H NMR (400 MHz, CDCl_3_) *δ* 6.44 (d, *J* = 9.0 Hz, 1H), 5.49–5.27 (m, 2H), 4.71 (d, *J* = 6.8 Hz, 1H), 4.61 (d, *J* = 6.8 Hz, 1H), 4.40 (dd, *J* = 9.1, 5.3 Hz, 1H), 4.04–3.91 (m, 1H), 3.72 (dd, *J* = 7.8, 5.3 Hz, 1H), 3.40 (s, 3H), 2.16–1.85 (m, 4H), 1.65–1.45 (m, 3H), 1.44–1.18 (m, 29H), 0.87 (t, *J* = 6.7 Hz, 3H); ^13^C NMR (100 MHz, CDCl_3_) *δ* 135.7, 130.1, 130.0, 109.4, 94.6, 93.8, 82.0, 77.1, 76.2, 56.0, 33.9, 32.1, 30.0, 29.9, 29.81, 29.76, 29.7, 29.6, 29.54, 29.51, 29.4, 27.7, 27.4, 26.9, 26.2, 22.9, 14.3; HRMS (ESI) calcd for C_28_H_50_Br_2_O_4_K^+^ [M + K]^+^ 647.1707, found 647.1710.

### Synthesis of compound 17a

To a stirred solution of the dibromoalkene 16a (65 mg, 0.10 mmol) in 6 mL anhydrous THF was added 60% sodium hydride (42 mg, 1.05 mmol) as a solid at 0 °C. The resulting mixture was warmed to room temperature, added two drops of water slowly and stirred overnight. After the reaction mixture was quenched with saturated aq. NH_4_Cl, it was extracted with EtOAc. The combined organic layers were dried over Na_2_SO_4_, filtered, and finally evaporated under a reduced pressure to give the crude 1-bromoalkyne 6a, which was used for the next reaction without further purification.

To a stirred aqueous solution of 30% *n*-BuNH_4_ (4 mL) was added crystal CuCl at room temperature, resulted in the formation of a blue solution immediately. A few crystals of hydroxylamine hydrochloride were added until the blue color disappeared. The resulting colorless solution indicated the present of Cu(i) salt. A solution of propargyl alcohol (0.12 mL, 2.09 mmol) in Et_2_O (0.5 mL) was added to the solution at room temperature, yielding a yellow acetylide suspension, which was immediately cooled to 0 °C. A solution of 1-bromoalkyne 6a in Et_2_O (0.5 mL) was added dropwise. The resulting mixture was warmed to room temperature and stirred for 4 h. More crystals of hydroxylamine hydrochloride were added throughout the reaction to prevent the reaction mixture from turning blue or green. The reaction mixture was extracted with EtOAc. The combined organic layers were dried over Na_2_SO_4_, filtered, and finally evaporated under a reduced pressure. The residue was purified by chromatography on silica gel (petroleum ether/EtOAc 6 : 1) to afford 17a as a colorless oil. Yield: 37 mg (69% for two steps): [*α*]^25^_D_ = −79.5 (*c* 0.4, CHCl_3_); ^1^H NMR (400 MHz, CDCl_3_) *δ* 5.45–5.29 (m, 2H), 4.92 (d, *J* = 6.8 Hz, 1H), 4.64 (d, *J* = 6.8 Hz, 1H), 4.52 (d, *J* = 6.3 Hz, 1H), 4.34 (s, 2H), 4.03 (td, *J* = 7.9, 3.2 Hz, 1H), 3.80 (t, *J* = 6.9 Hz, 1H), 3.40 (s, 3H), 2.09–1.85 (m, 4H), 1.82–1.68 (m, 1H), 1.63–1.46 (m, 5H), 1.45–1.22 (m, 21H), 1.21–1.10 (m, 2H), 0.86 (d, *J* = 6.5 Hz, 6H); ^13^C NMR (100 MHz, CDCl_3_) *δ* 130.2, 130.0, 109.7, 94.5, 81.6, 78.6, 77.7, 75.4, 71.5, 69.8, 67.7, 56.0, 51.6, 39.2, 34.2, 30.01, 29.99, 29.97, 29.7, 29.62, 29.56, 29.5, 28.2, 27.8, 27.6, 27.4, 27.2, 26.1, 22.9; HRMS *m*/*z* (ESI) calcd for C_31_H_53_O_5_^+^ [M + H]^+^ 505.3888, found 505.3877.

### Synthesis of compound 17b

To a stirred solution of dibromoalkene 16b (110 mg, 0.18 mmol) in 20 mL anhydrous THF was added 60% sodium hydride (211 mg, 3.53 mmol) as a solid at 0 °C. The resulting mixture was warmed to room temperature, added two drops of water slowly and stirred overnight. After the reaction mixture was quenched with saturated aq. NH_4_Cl, it was extracted with EtOAc. The combined organic layers were dried over Na_2_SO_4_, filtered, and finally evaporated under a reduced pressure to give the crude 1-bromoalkyne 6b, which was used for the next reaction without further purification.

To a stirred 30% *n*-BuNH_4_ aqueous solution (6 mL) was added crystal of CuCl at room temperature, resulted in the formation of a blue solution immediately. A few crystal of hydroxylamine hydrochloride were added until the blue color disappeared. The resulting colorless solution indicated the present of Cu(i) salt. A solution of propargyl alcohol (0.17 mL, 3.53 mmol) in Et_2_O (0.5 mL) was added to the solution at room temperature, yielding a yellow acetylide suspension, which was immediately cooled to 0 °C. A solution of 1-bromoalkyne 6b in Et_2_O (0.5 mL) was added dropwise. The resulting mixture was warmed to room temperature and stirred for 4 h. More crystals of hydroxylamine hydrochloride were added throughout the reaction to prevent the reaction mixture from turning blue or green. The reaction mixture was extracted with EtOAc. The combined organic layers were dried over Na_2_SO_4_, filtered, and finally evaporated under a reduced pressure. The residue was purified by chromatography on silica gel (petroleum ether/EtOAc 6 : 1) to afford 17b as a colorless oil. Yield: 53 mg (58% for two steps): [*α*]^25^_D_ = −114.0 (*c* 0.2, CHCl_3_); ^1^H NMR (400 MHz, CDCl_3_) *δ* 5.45–5.27 (m, 2H), 4.92 (d, *J* = 6.8 Hz, 1H), 4.64 (d, *J* = 6.8 Hz, 1H), 4.52 (d, *J* = 6.3 Hz, 1H), 4.33 (s, 2H), 4.03 (td, *J* = 7.9, 3.3 Hz, 1H), 3.79 (dd, *J* = 7.4, 6.4 Hz, 1H), 3.39 (s, 3H), 2.12–1.87 (m, 4H), 1.83–1.68 (m, 2H), 1.65–1.46 (m, 2H), 1.44–1.23 (m, 28H), 0.93–0.82 (d, *J* = 6.8 Hz, 3H); ^13^C NMR (100 MHz, CDCl_3_) *δ* 130.2, 130.0, 109.7, 94.5, 81.6, 78.5, 77.7, 75.3, 71.5, 69.7, 67.7, 56.0, 51.6, 34.2, 32.1, 29.97, 29.95, 29.80, 29.75, 29.7, 29.6, 29.53, 29.51, 29.45, 27.8, 27.4, 27.1, 26.1, 22.9, 14.3; HRMS *m*/*z* (ESI) calcd for C_31_H_52_O_5_Na^+^ [M + Na]^+^ 527.3707, found 527.3706.

### Synthesis of petrosiol B (2)

To a stirred solution of diyne 17a (25 mg, 0.048 mmol) in 3 mL EtOH was added 3 N HCl (0.5 mL) aqueous solution at room temperature. The mixture was stirred at 70 °C for 1.5 h. The reaction mixture was cooled to room temperature and evaporated under a reduced pressure to afford 2 as a white powder. Yield: 20 mg (98%): [*α*]^25^_D_ = −4.2 (*c* 0.25, MeOH); ^1^H NMR (400 MHz, CDCl_3_/MeOD = 4 : 1) *δ* 5.45–5.27 (m, 2H), 4.46 (d, *J* = 6.9 Hz, 1H), 4.27 (s, 2H), 3.82–3.68 (m, 1H), 3.44 (dd, *J* = 6.9, 2.2 Hz, 1H), 2.13–1.88 (m, 4H), 1.69–1.40 (m, 4H), 1.38–1.23 (m, 17H), 1.21–1.09 (m, 2H), 0.87 (d, *J* = 6.6 Hz, 6H); ^1^H NMR (400 MHz, MeOD) *δ* 5.46–5.26 (m, 2H), 4.43 (d, *J* = 7.6 Hz, 1H), 4.24 (s, 2H), 3.83–3.67 (m, 1H), 3.37 (dd, *J* = 7.6, 2.3 Hz, 1H), 2.14–1.91 (m, 4H), 1.66–1.45 (m, 4H), 1.41–1.24 (m, 17H), 1.23–1.13 (m, 2H), 0.89 (d, *J* = 6.6 Hz, 6H); ^13^C NMR (100 MHz, CDCl_3_/MeOD = 4 : 1) *δ* 130.2, 130.1, 78.4, 77.6, 76.2, 71.3, 70.2, 69.0, 64.5, 50.7, 39.3, 34.3, 30.07, 30.06, 30.0, 29.9, 29.8, 29.59, 29.58, 28.2, 27.6, 27.47, 27.46, 26.0, 22.8; ^13^C NMR (100 MHz, MeOD) *δ* 130.9, 130.8, 79.3, 79.2, 77.7, 72.1, 70.6, 69.4, 65.4, 51.0, 40.2, 35.0, 30.9, 30.84, 30.77, 30.7, 30.39, 30.35, 29.2, 28.5, 28.2, 28.1, 26.9, 23.1; HRMS (ESI) calcd for C_26_H_44_O_4_K^+^ [M + K]^+^ 459.2871, found 459.2865.

### Synthesis of petrosiol D (4)

To a stirred solution of diyne 17b (19 mg, 0.037 mmol) in 4 mL EtOH was added 3 N HCl (0.6 mL) aqueous solution at room temperature. The mixture was stirred at 70 °C for 2 h. The reaction mixture was cooled to room temperature and evaporated under a reduced pressure to afford 4 as a white powder. Yield: 15 mg (98%): [*α*]^25^_D_ = −4.8 (*c* 0.18, MeOH); ^1^H NMR (400 MHz, CDCl_3_/MeOD = 4 : 1) *δ* 5.44–5.27 (m, 2H), 4.46 (d, *J* = 6.7 Hz, 1H), 4.27 (s, 2H), 3.82–3.71 (m, 1H), 3.45 (d, *J* = 6.3 Hz, 1H), 2.08–1.92 (m, 4H), 1.68–1.42 (m, 3H), 1.37–1.18 (m, 23H), 0.93–0.82 (d, *J* = 6.7 Hz, 3H); ^1^H NMR (400 MHz, MeOD) *δ* 5.55–5.25 (m, 2H), 4.44 (d, *J* = 7.6 Hz, 1H), 4.24 (s, 2H), 3.82–3.70 (m, 1H), 3.38 (dd, *J* = 7.6, 2.3 Hz, 1H), 2.14–1.93 (m, 4H), 1.67–1.42 (m, 3H), 1.41–1.24 (m, 23H), 0.95–0.85 (d, *J* = 6.7 Hz, 3H); ^13^C NMR (100 MHz, CDCl_3_/MeOD = 4 : 1) *δ* 130.1, 130.0, 78.3, 77.6, 76.1, 71.3, 70.1, 68.9, 64.4, 50.6, 34.2, 32.1, 30.0, 29.9, 29.81, 29.76, 29.7, 29.50, 29.47, 29.46, 27.37, 27.35, 25.9, 22.8, 14.2; ^13^C NMR (100 MHz, MeOD) *δ* 130.8 (2C), 79.3, 79.2, 77.6, 72.1, 70.6, 69.4, 65.3, 51.0, 35.0, 33.1, 30.9, 30.83, 30.75, 30.73, 30.65, 30.6, 30.5, 30.4, 30.3, 28.2, 28.1, 26.9, 23.8, 14.5; HRMS (ESI) calcd for C_26_H_44_O_4_Na^+^ [M + Na]^+^ 443.3132, found 443.3134.

### Bioactivity section

#### PC12 cell culture and neuronal differentiation

PC12 cell was obtained from the Shanghai Cell Bank of Type Culture Collection of Chinese Academy of Science. Cells were maintained in DMEM (Gibco, Life Technologies) containing 6% (v/v) fetal bovine serum (FBS, Gibco, Life Technologies), 6% (v/v) horse serum (HS, Gibco, Life Technologies) and 100 U mL^−1^ penicillin–streptomycin (Gibco, Life Technologies). Cells were incubated under a humidified atmosphere of 95% O_2_ and 5% CO_2_ at 37 °C. For differentiation experiments, PC12 cells were cultured in differentiation medium: DMEM medium with 1% HS, 1% FBS, and 100 U mL^−1^ penicillin–streptomycin. Petrosiol B, D and E were dissolved in dimethyl sulfoxide (DMSO, Amresco). The medium was replaced by a differentiation medium containing 0.1% DMSO and the compounds (petrosiol B, D and E at the indicated concentrations) when the cells reached 20–30% confluency. We previously proved that petrosiol E, under 5 μg mL^−1^, induced the differentiation of PC12 by enhancing Nrf2 activity, thus we used petrosiol E as positive control in this study. Medium containing 0.1% DMSO did not elicit any toxicity to cells or change the ability of cell differentiation. Thus, medium containing 0.1% DMSO served as the vehicle control in this paper.

#### Cell viability assay

Cell viability was determined through the CCK-8 assay (CCK8, Dojindo Laboratories). First, PC12 cells were seeded in 96-well plates with 6 × 10^3^ per well overnight. Then, cells were cultured in differentiation medium containing petrosiol B, D and E at various concentrations for 1, 3 and 5 days, followed by the addition of 10 μL CCK-8 solution for 1 h. The absorbance of each well was measured at 450 nm using a microplate reader (Synergy 2, BioTek).

#### Assessment of neurite bearing cells

Neurite outgrowth was evaluated by the percentage of neurite-bearing cells. Morphological changes of PC12 cells were observed and photographed using EVOS XL Core Imaging System (Life Technologies) at different treated time. The cells were defined as neurite bearing cells when their neurites were >2 cell-body in length. In this study, ≈500 cells were screened in six randomly fields in each well. Lastly, the percentage of neurite-bearing cells was calculated by normalizing to the total number of cells in each field.

#### RNA extraction and qRT-PCR analysis

Total RNAs were extracted from cells using RNA extraction kit (BioTeke) and reverse transcribed into cDNA (TransGen). Quantitative reverse transcription polymerase chain reaction (qRT-PCR) was performed using SYBR Green PCR mix (Roche) with specific primers. The relative signal intensity was measured using a Real-Time PCR Detection System (ABI 7500). The primers used in the study were as follows: Rat HO-1 (forward, 5′-TGCTCGCATGAACACTCTG-3′; reverse, 5′-TCCTCTGTCAGCAGTGCCT-3′); Rat Gapdh (forward, 5′-AACCTGCCAAGTATGATGAC-3′; reverse, 5′-GGAGTTGCTGTTGAAGTCA-3′). Values were normalized against that of the housekeeping gene glyceraldehyde 3-phosphate dehydrogenase (Gapdh). Relative transcript expression was determined using a control sample as a calibrator and the ΔΔ*C*_*t*_ method. Data were presented as fold change relative to the vehicle control group.

#### Western blotting analysis

Cells after treatment were washed twice with PBS and nuclear proteins were extracted from cells using nuclear protein extraction kit (Solarbio). Then, equal amounts of proteins were subjected to 8–12% SDS-PAGE and western blot analysis. The primary antibodies used in this study: anti-Nrf2 Ab (16396-1-AP, 1 : 1000; Proteintech) and anti-Lamin A + C (ab169532, 1 : 5000; Abcam). Anti-Lamin A + C antibodies were used for equal loading of nuclear proteins.

#### Statistical analysis

Results are expressed as means ± SD of three independent experiments, unless otherwise specified. Significant differences were evaluated using one-way analysis of covariance (ANOVA) for multiple treatment groups. Values at *P* < 0.05 were considered significant.

## Conflicts of interest

There are no conflicts to declare.

## Supplementary Material

RA-009-C9RA01166H-s001
