# Supplementary material for: Synthesis and biological activities of petrosiols B and D
Source: RSC Adv. 2019 Apr 1;9(18):10253–63. doi: 10.1039/c9ra01166h (PMC9062523; doi:10.1039/c9ra01166h)
Supplement: RA-009-C9RA01166H-s001 [file RA-009-C9RA01166H-s001.pdf]

Electronic Supplementary Material (ESI) for RSC Advances.
This journal is © The Royal Society of Chemistry 2019

Supporting Information

Synthesis and biological activities of petrosiols B and D

Jialin Geng, [a,b] Qidong Ren, [a,b] Caizhu Chang,[a,c] Xinni Xie, [a] Jun Liu,*[a, b] and Yuguo Du*[a,b,d]

[a]State Key Laboratory of Environmental Chemistry and Eco-toxicology, Research Center for Eco-Environmental Sciences, Chinese Academy of Science, Beijing 100085, China;

[b]School of Chemical Sciences, University of Chinese Academy of Sciences, Beijing 100049, China

[c]School of Chemistry and Environmental Engineering, Wuhan Institute of Technology, Wuhan 430205, China

[d]National Engineering Research Center for Carbohydrate Synthesis, Jiangxi Normal University, Nanchang 330022, China;

Contents:

^{1}H and ^{13}C NMR ($CDCl_3$) Spectra of Compound **10**--S3

^{1}H and ^{13}C NMR ($CDCl_3$) Spectra of Compound **11**--S4

^{1}H and ^{13}C NMR ($CDCl_3$) Spectra of Compound **12**--S5

^{1}H and ^{13}C NMR ($CDCl_3$) Spectra of Compound **7**--S6

^{1}H and ^{13}C NMR ($CDCl_3$) Spectra of Compound **13**--- -S7

^{1}H and ^{13}C NMR ($CDCl_3$) Spectra of Compound **14a**-- S8

^{1}H and ^{13}C NMR ($CDCl_3$) Spectra of Compound **14b**-- S9

^{1}H and ^{13}C NMR ($CDCl_3$) Spectra of Compound **15a**-- S10

^{1}H and ^{13}C NMR ($CDCl_3$) Spectra of Compound **15b**-- S11

^{1}H and ^{13}C NMR ($CDCl_3$) Spectra of Compound **16a**-- S12

^{1}H and ^{13}C NMR ($CDCl_3$) Spectra of Compound **16b**-- S13

^{1}H and ^{13}C NMR ($CDCl_3$) Spectra of Compound **17a**-- S14

^{1}H and ^{13}C NMR ($CDCl_3$) Spectra of Compound **17b**-- S15

^{1}H and ^{13}C NMR ($CDCl_3$/ MeOD =4:1) Spectra of Compound **2**----------------------------------- S16

^{1}H and ^{13}C NMR (MeOD) Spectra of Compound **2**--- S17

^{1}H and ^{13}C NMR ($CDCl_3$/ MeOD =4:1) Spectra of Compound **4**----------------------------------- S18

^{1}H and ^{13}C NMR (MeOD) Spectra of Compound **4**--- S19

Comparison of ^{1}H and ^{13}C NMR data of natural and synthetic petrosiol B (**2**) ------------------- S20

Comparison of ^{1}H and ^{13}C NMR data of natural and synthetic petrosiol D (**4**) ------------------- S21

^{1}H and ^{13}C NMR ($CDCl_3$) Spectra of Compound **10**

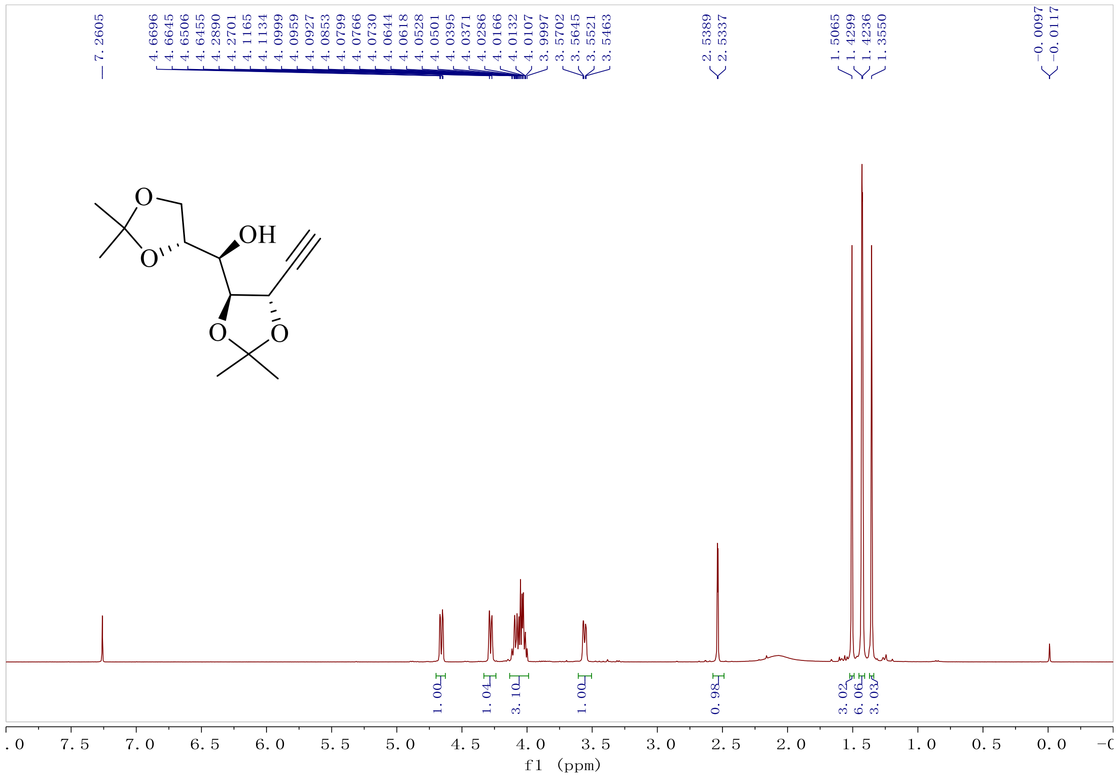

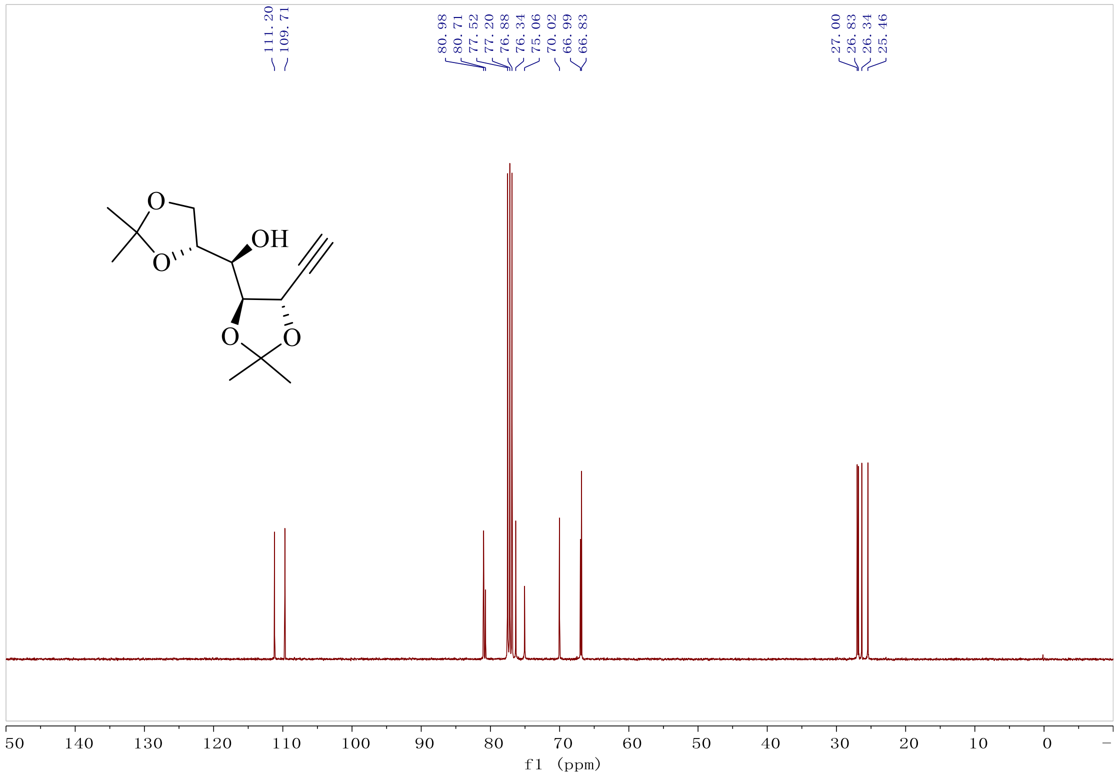

^{1}H and ^{13}C NMR ($CDCl_3$) Spectra of Compound **11**

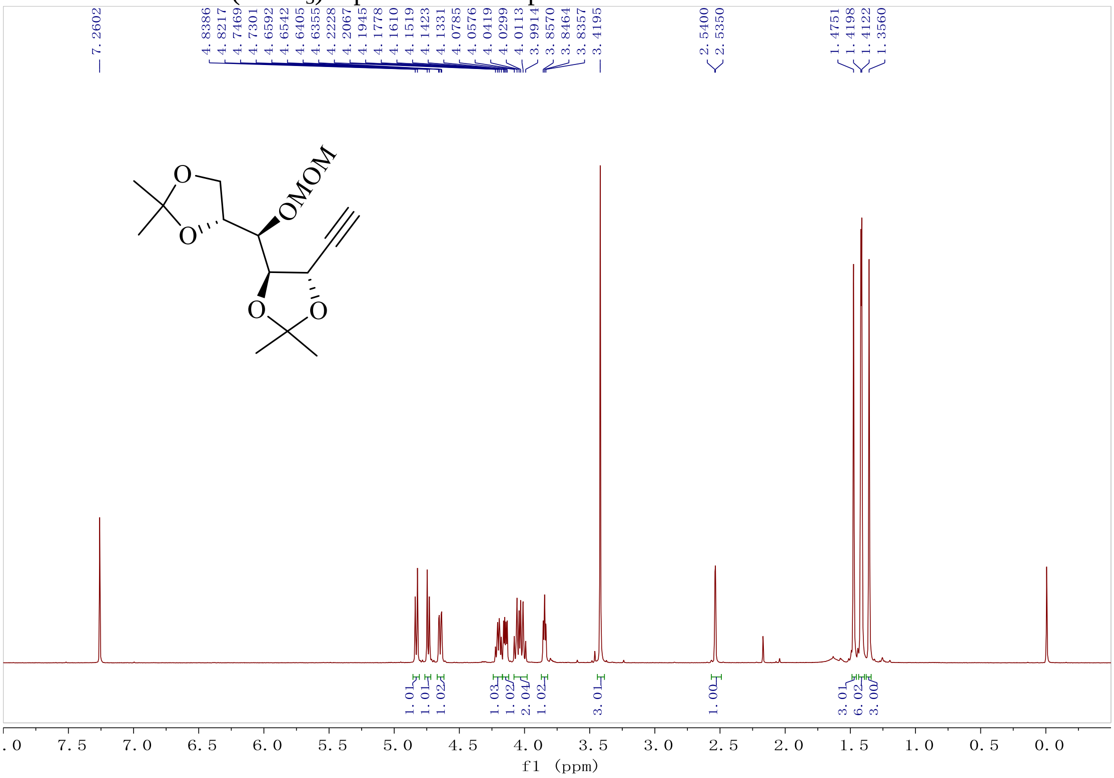

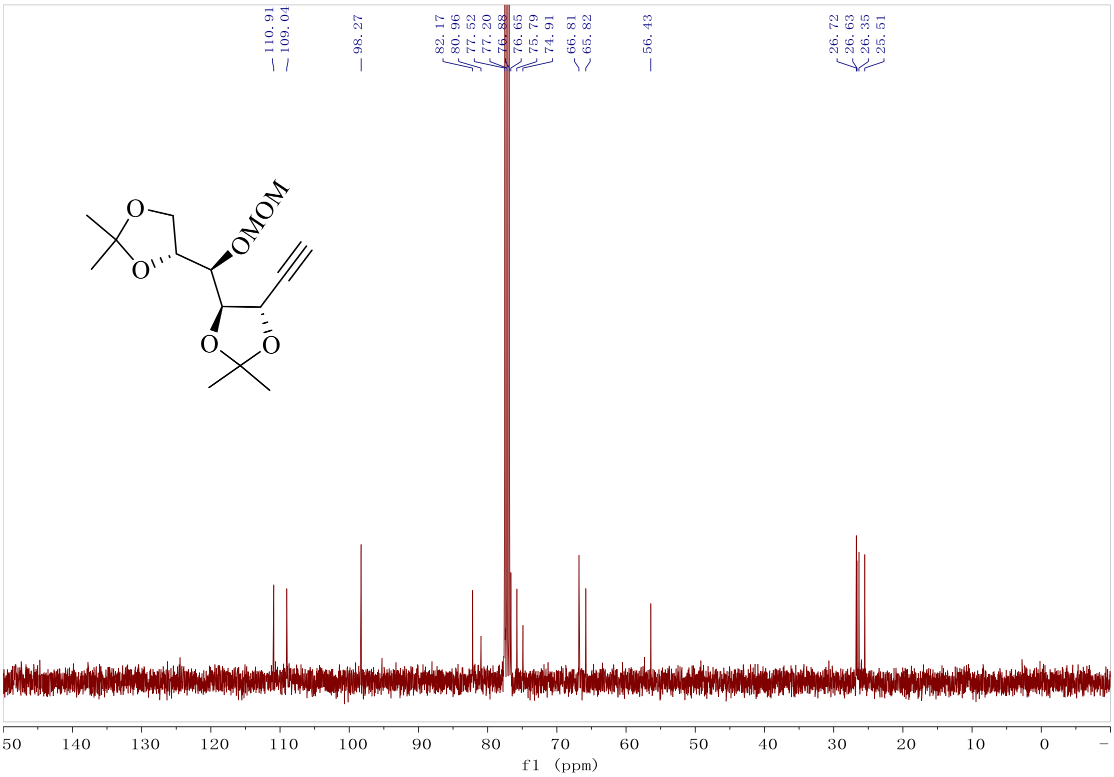

^{1}H and ^{13}C NMR ($CDCl_3$) Spectra of Compound **12**

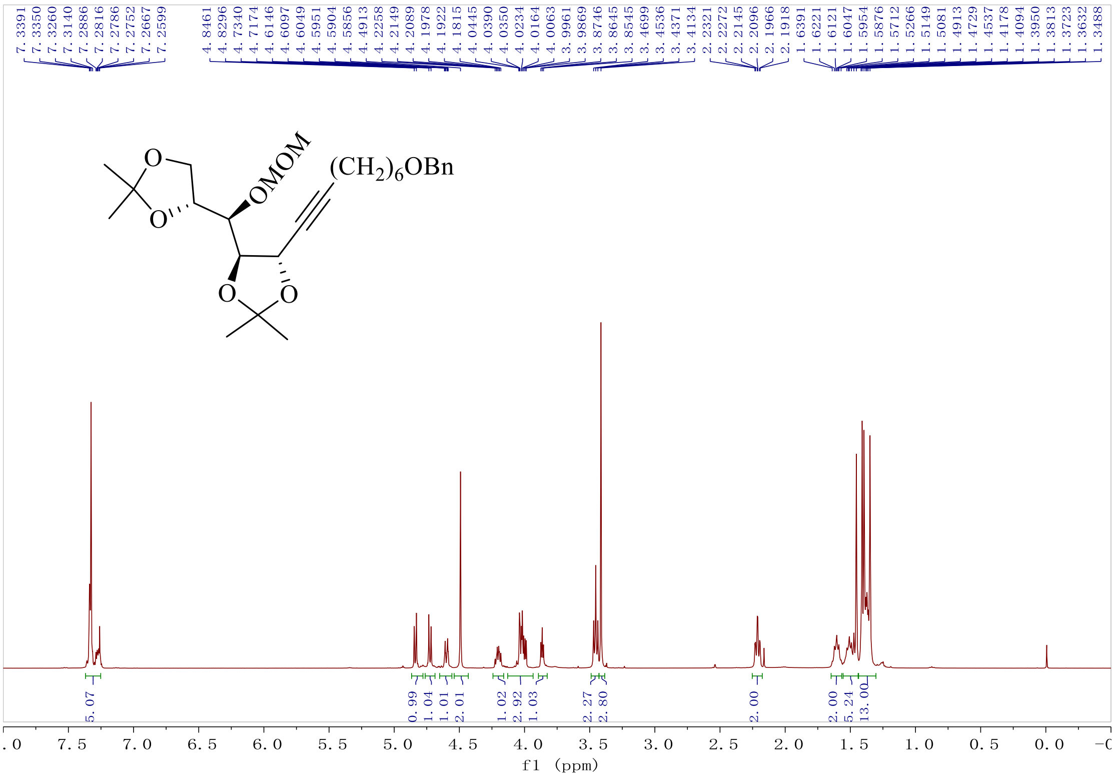

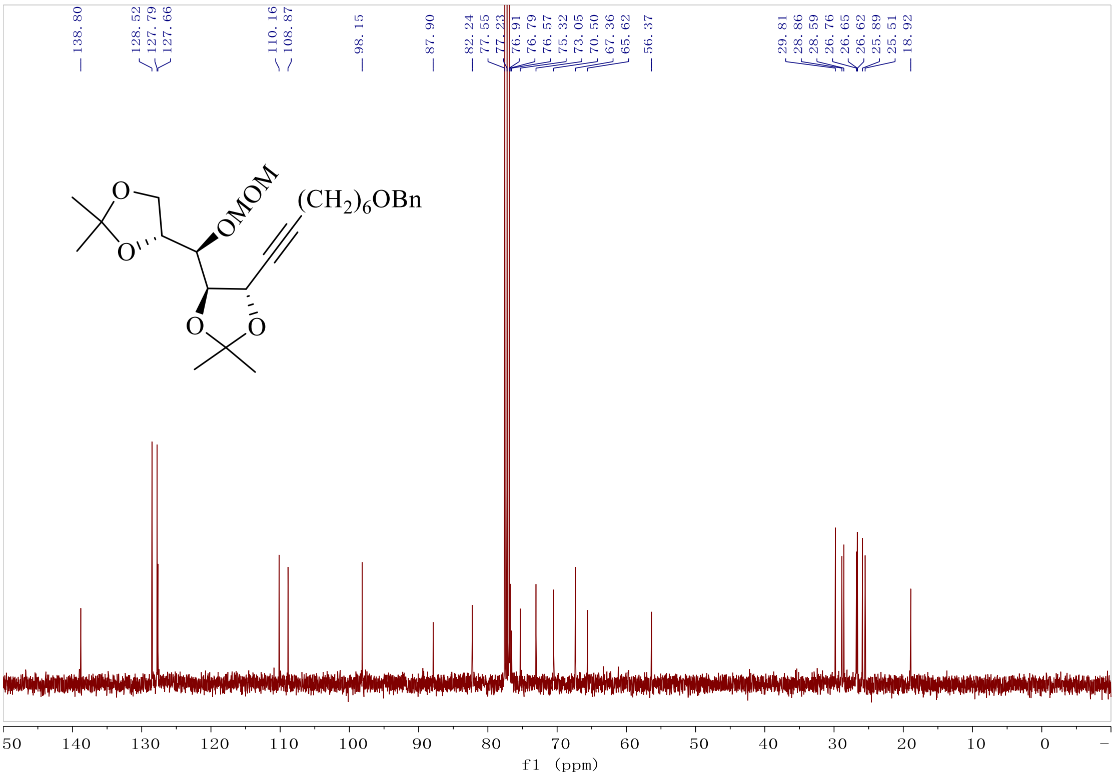

^{1}H and ^{13}C NMR ($CDCl_3$) Spectra of Compound **7**

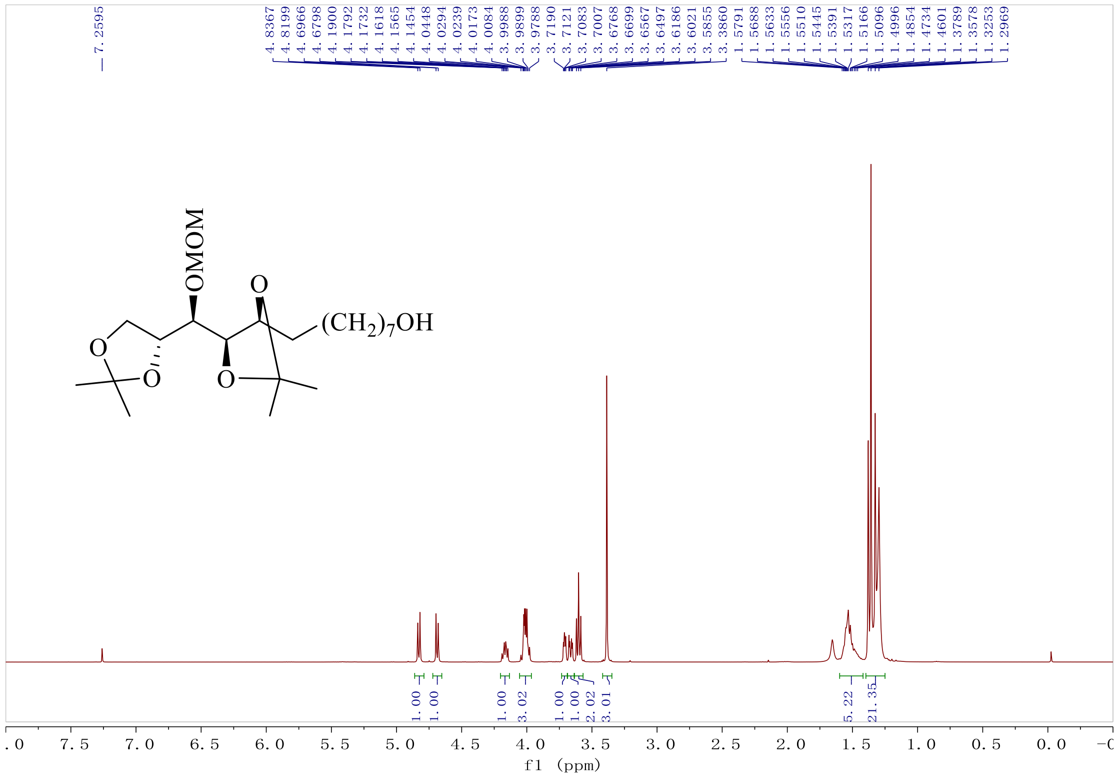

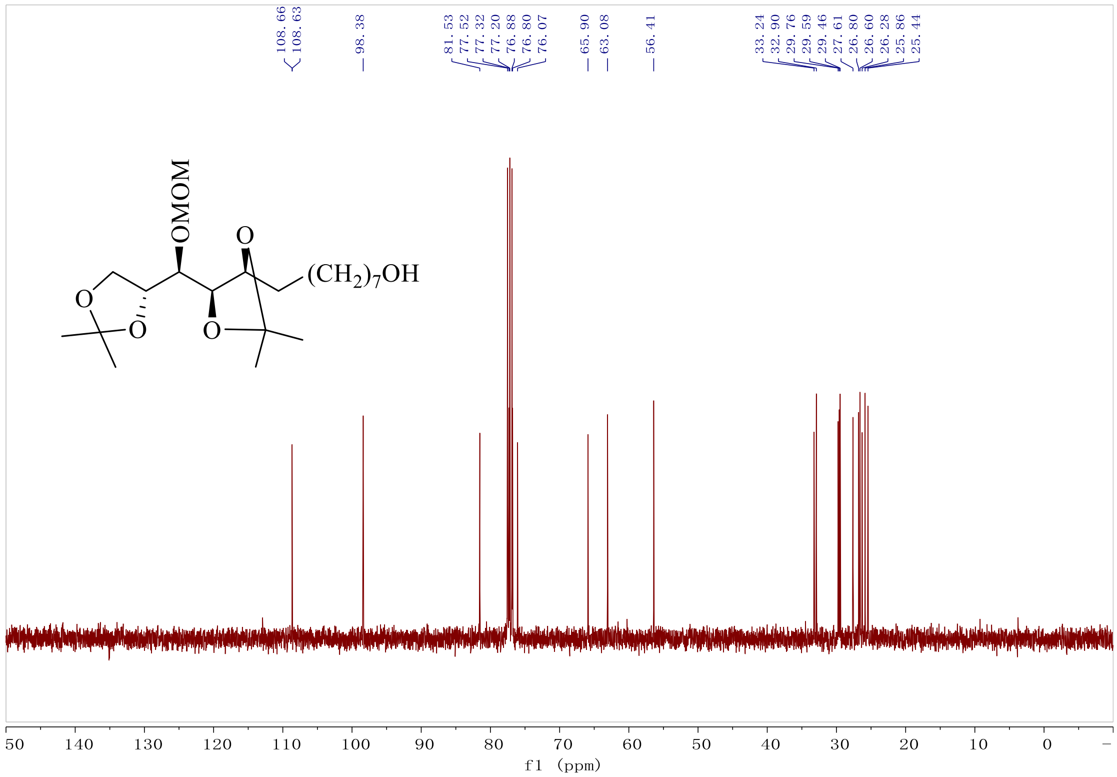

^{1}H and ^{13}C NMR ($CDCl_3$) Spectra of Compound **13**

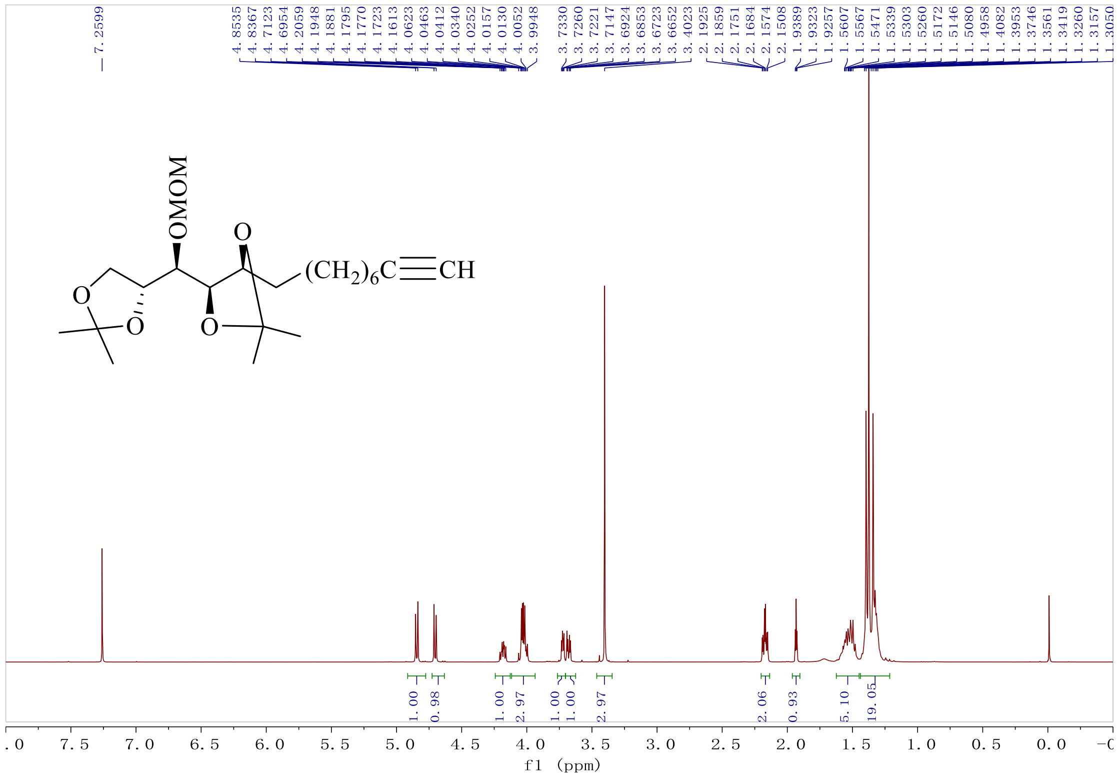

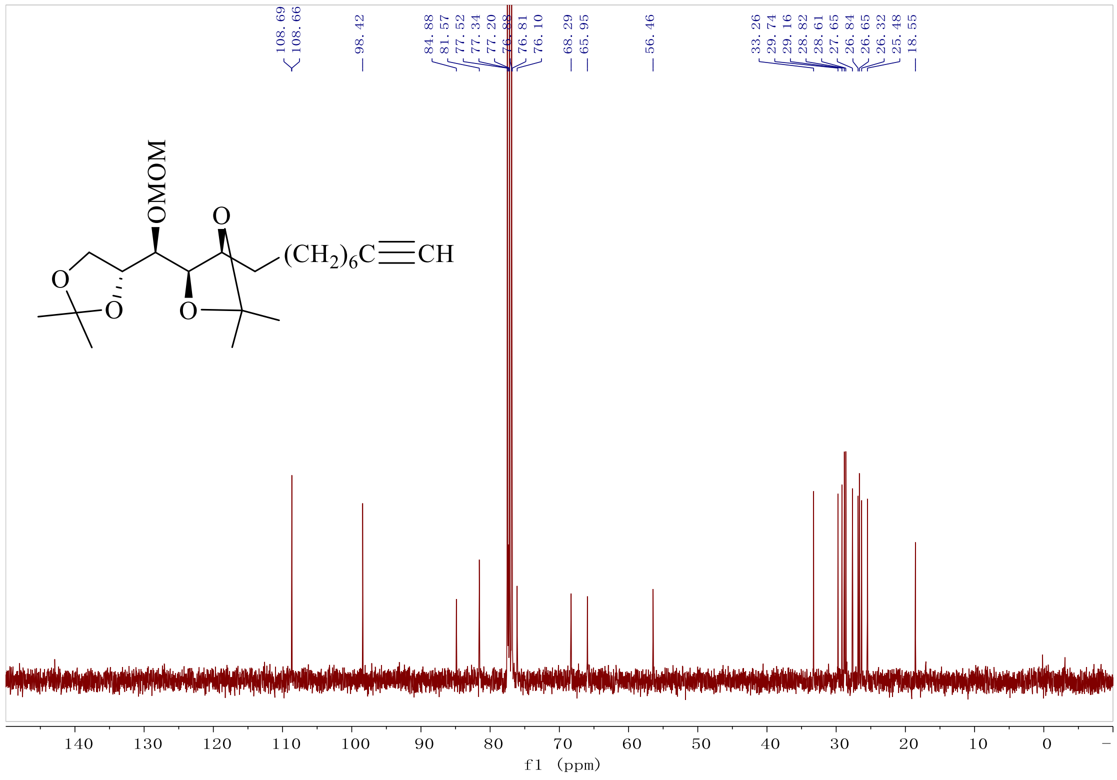

^{1}H and ^{13}C NMR ($CDCl_3$) Spectra of Compound **14a**

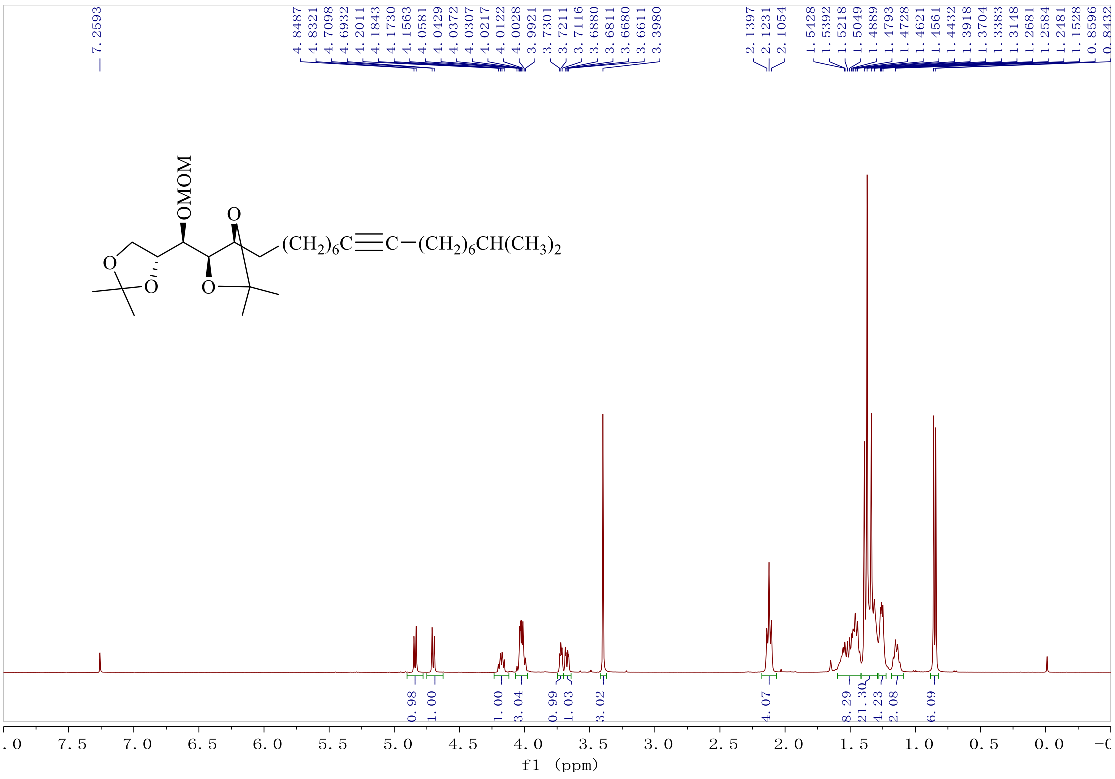

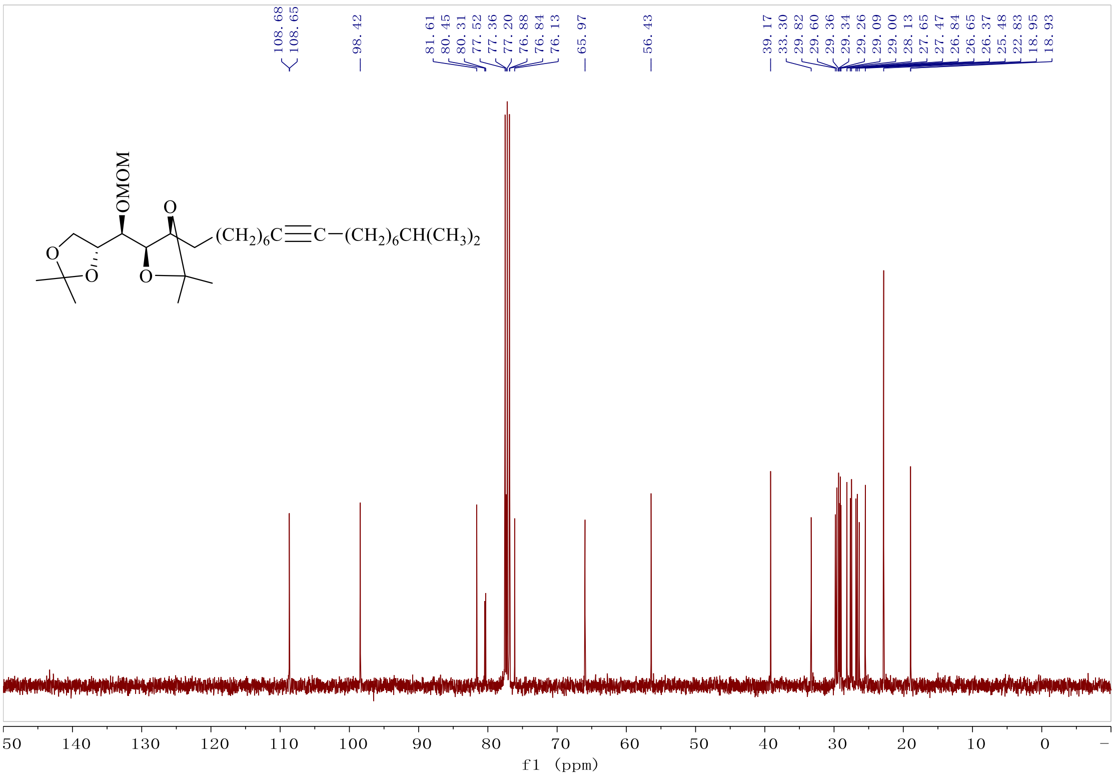

^{1}H and ^{13}C NMR ($CDCl_3$) Spectra of Compound **14b**

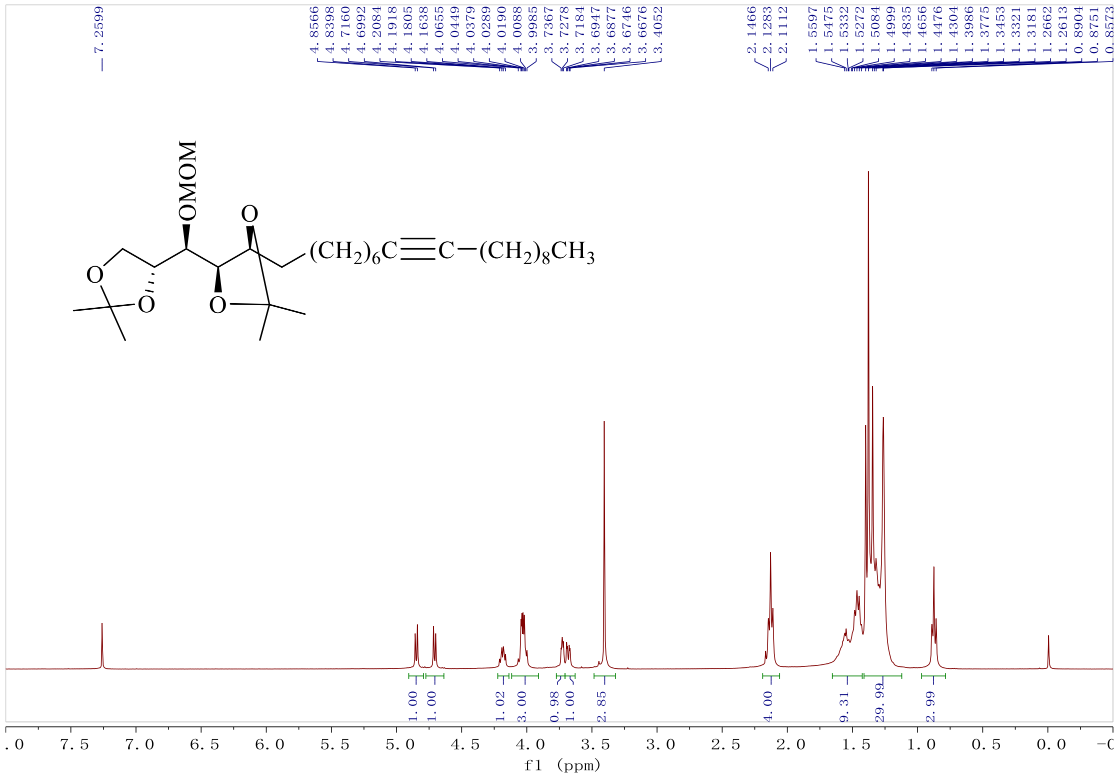

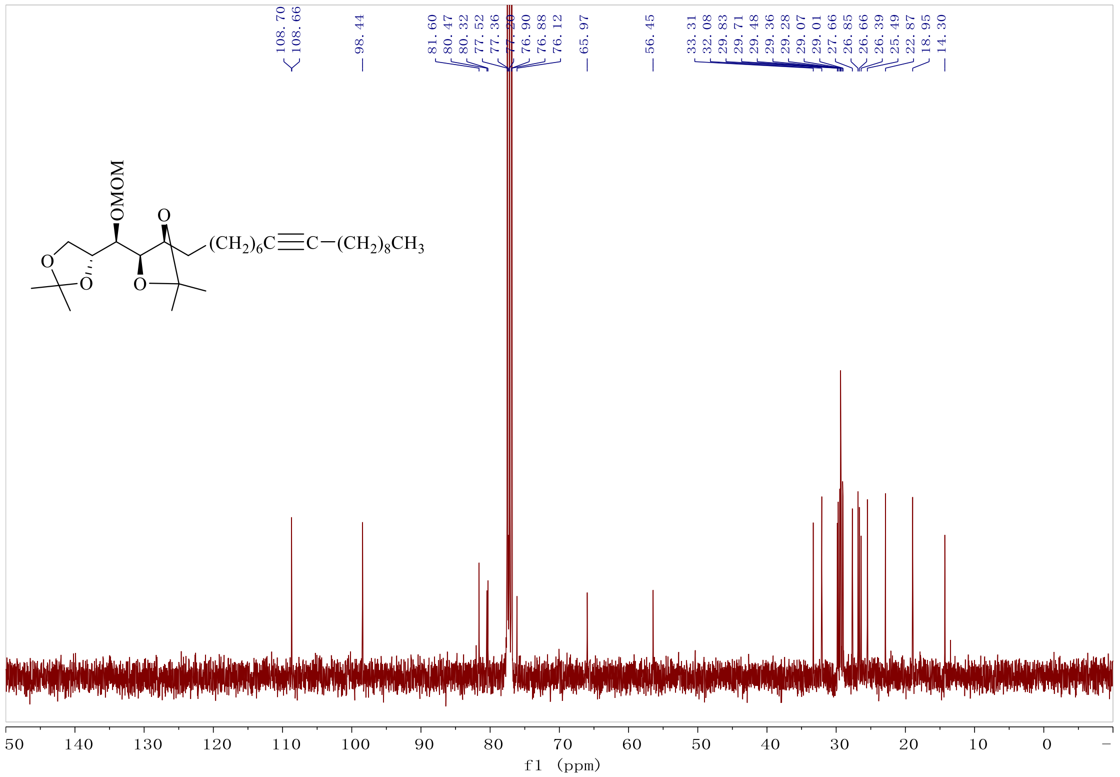

^{1}H and ^{13}C NMR ($CDCl_3$) Spectra of Compound **15a**

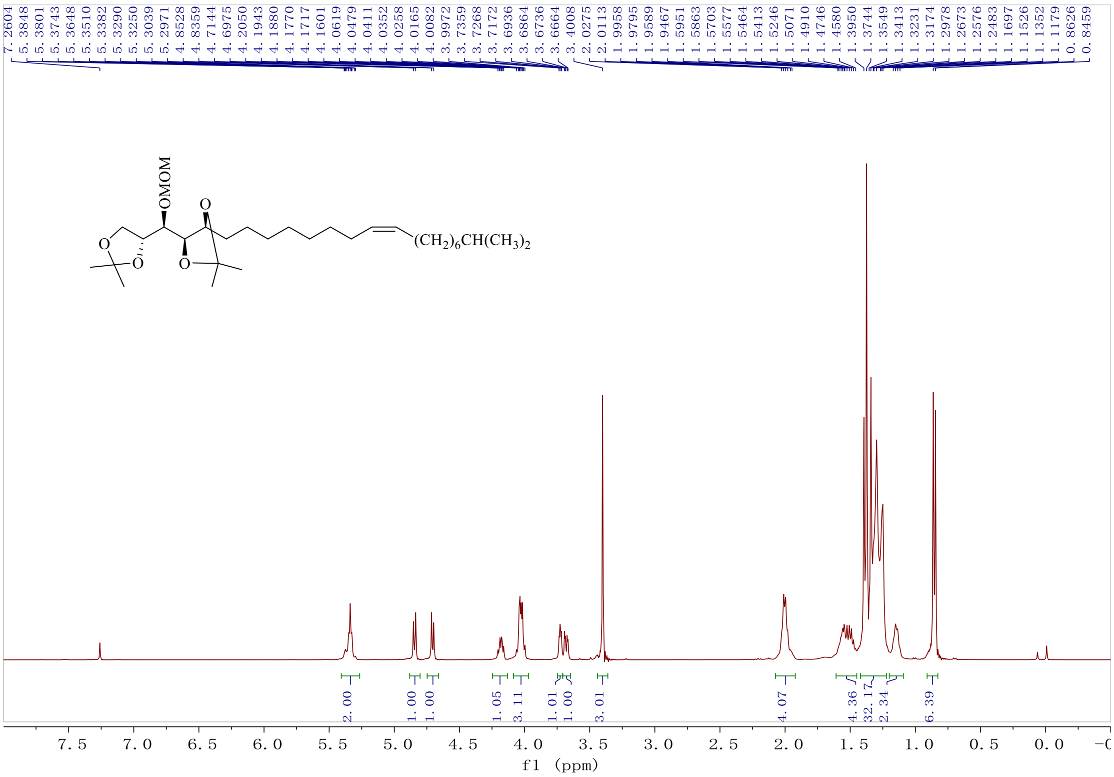

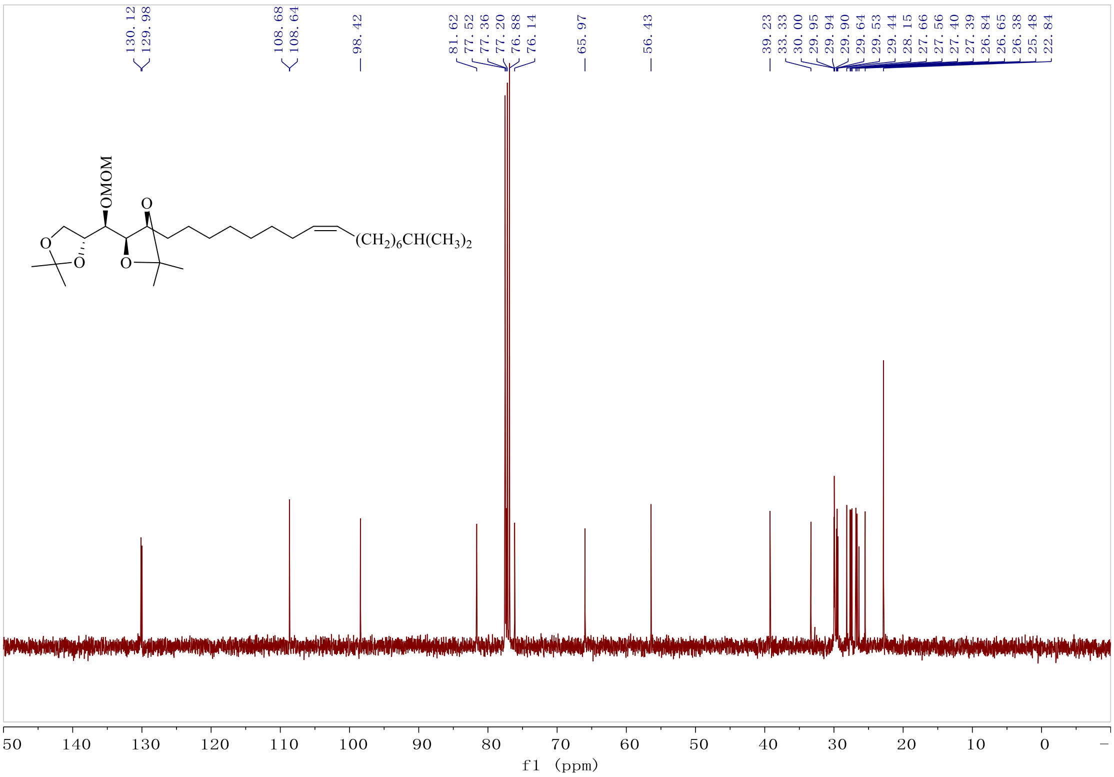

^{1}H and ^{13}C NMR ($CDCl_3$) Spectra of Compound **15b**

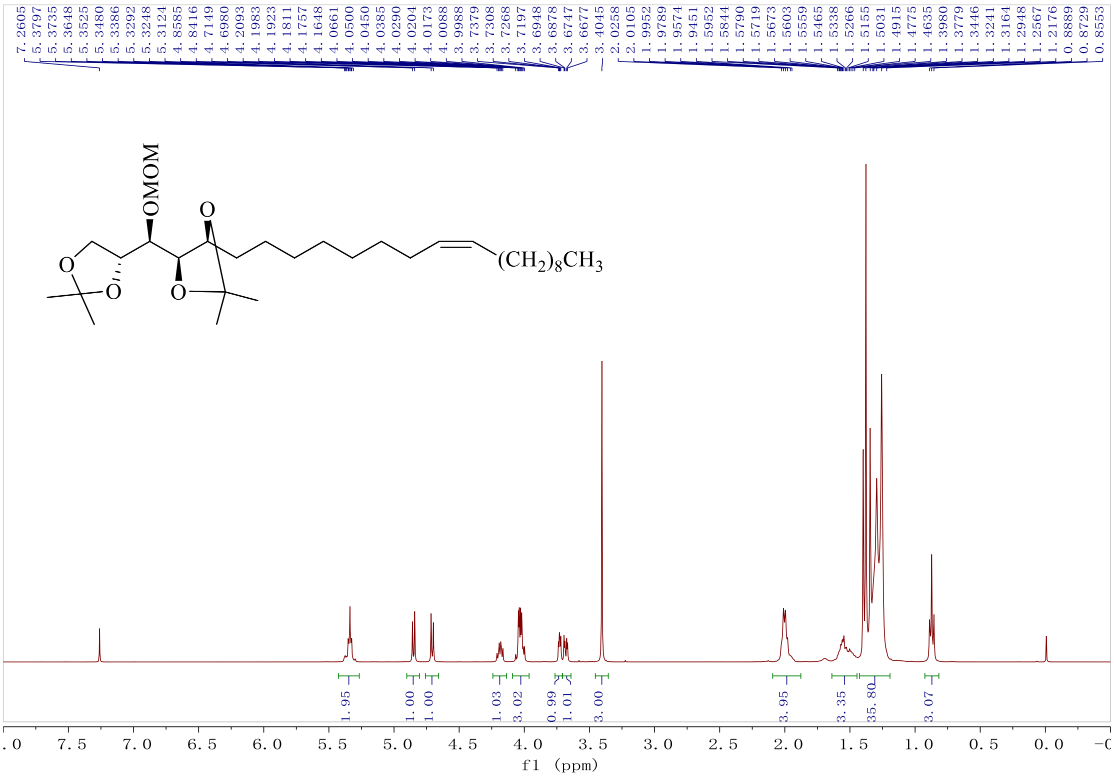

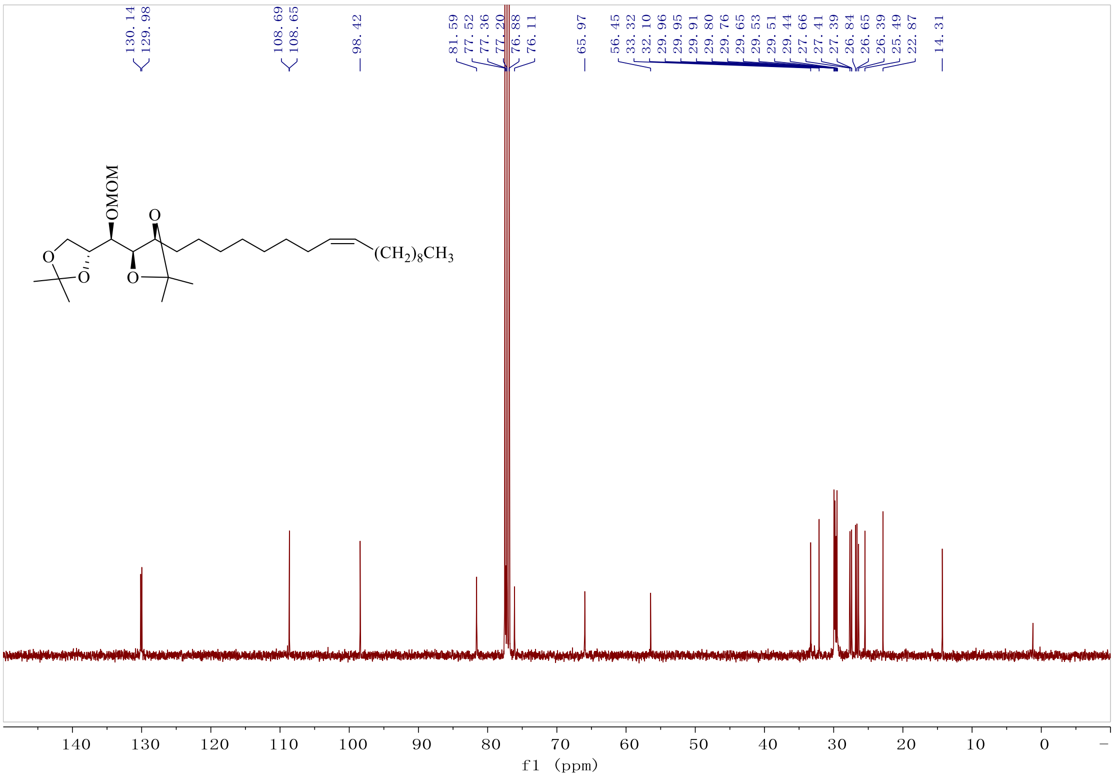

^{1}H and ^{13}C NMR ($CDCl_3$) Spectra of Compound **16a**

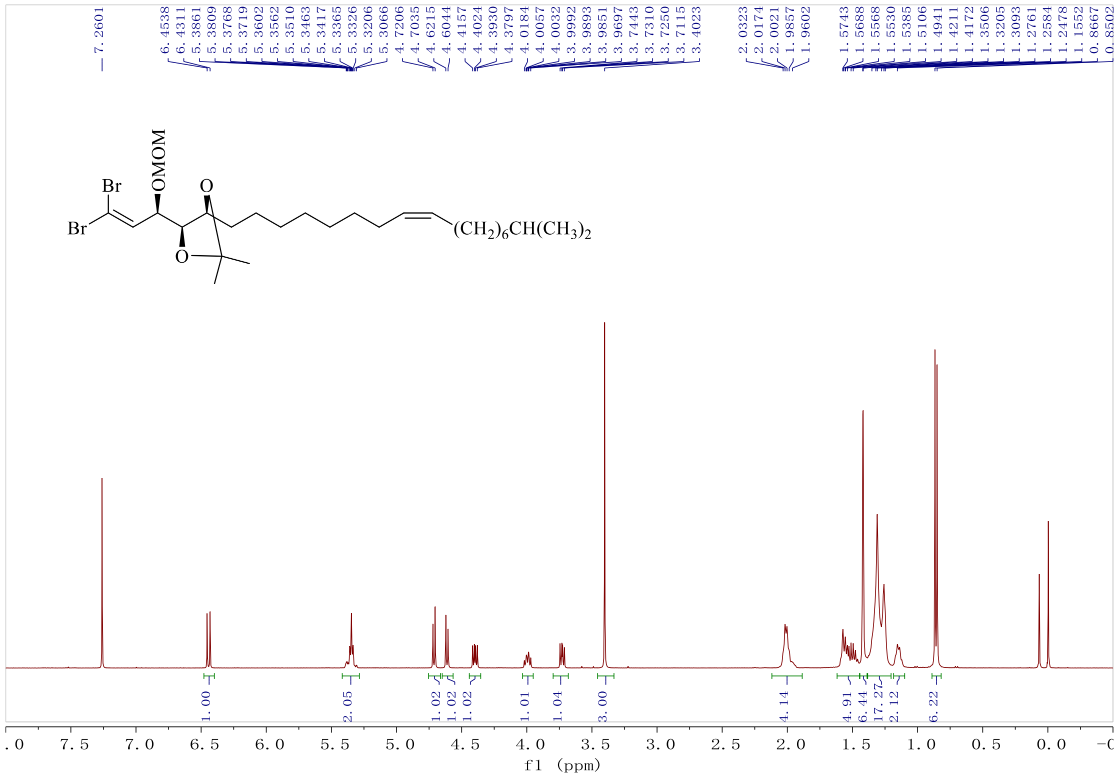

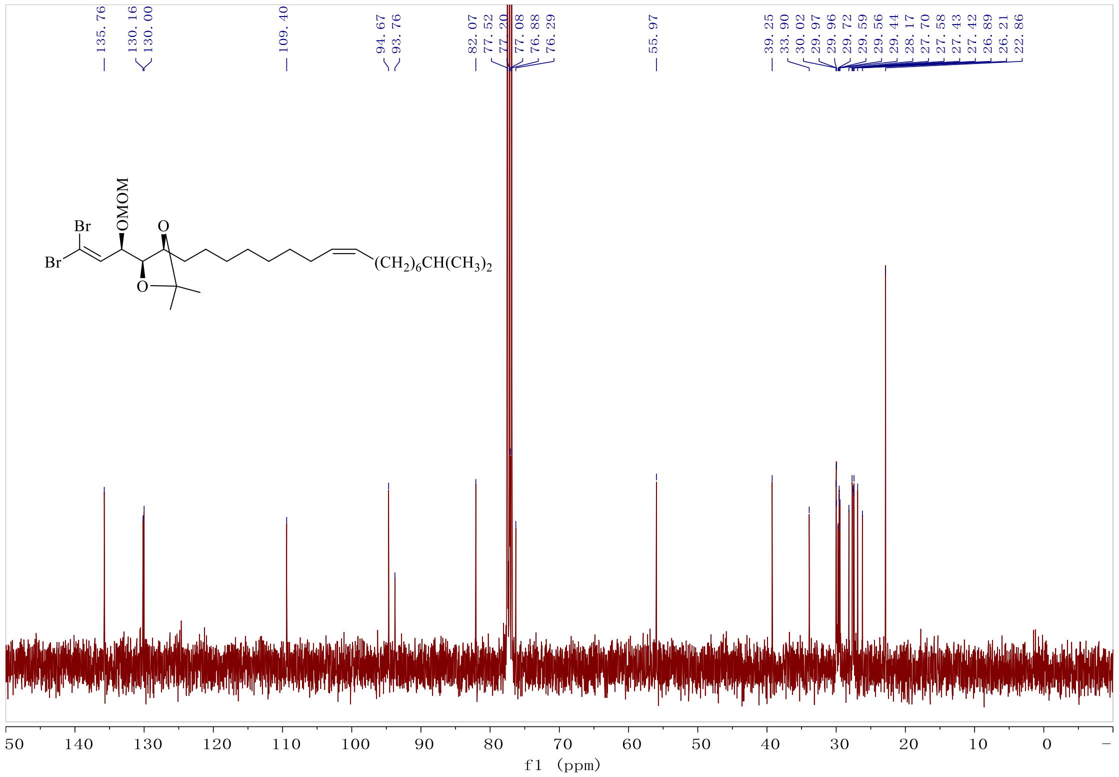

^{1}H and ^{13}C NMR ($CDCl_3$) Spectra of Compound **16b**

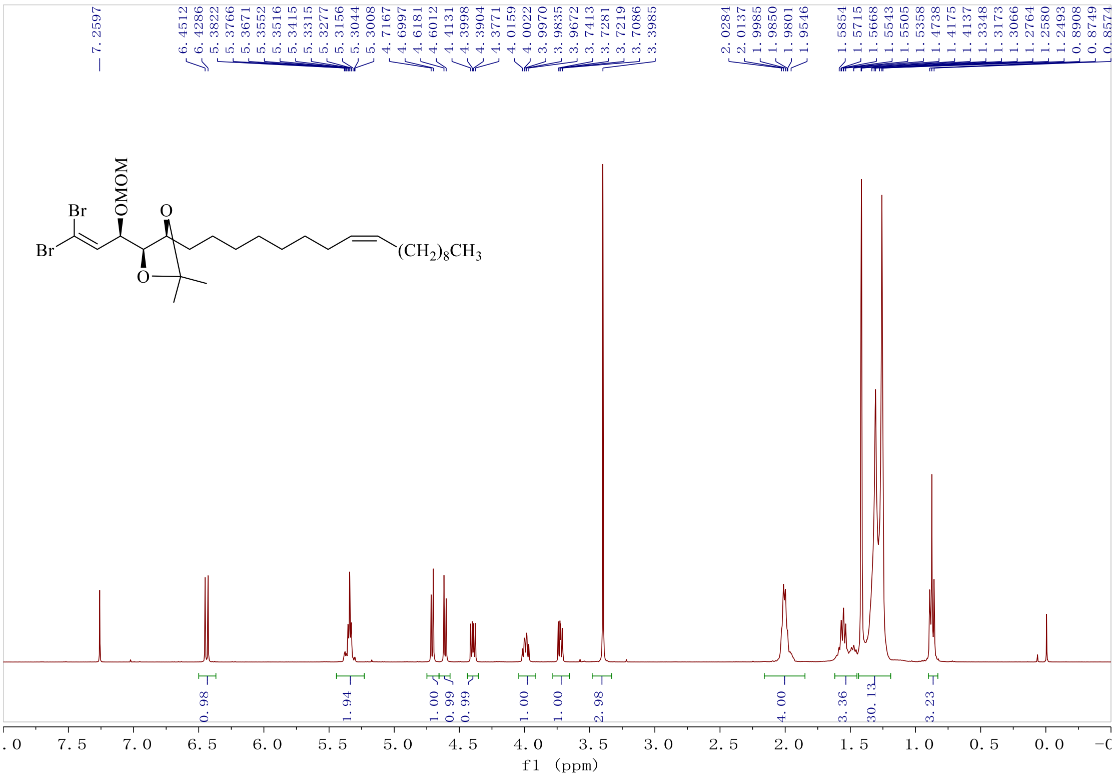

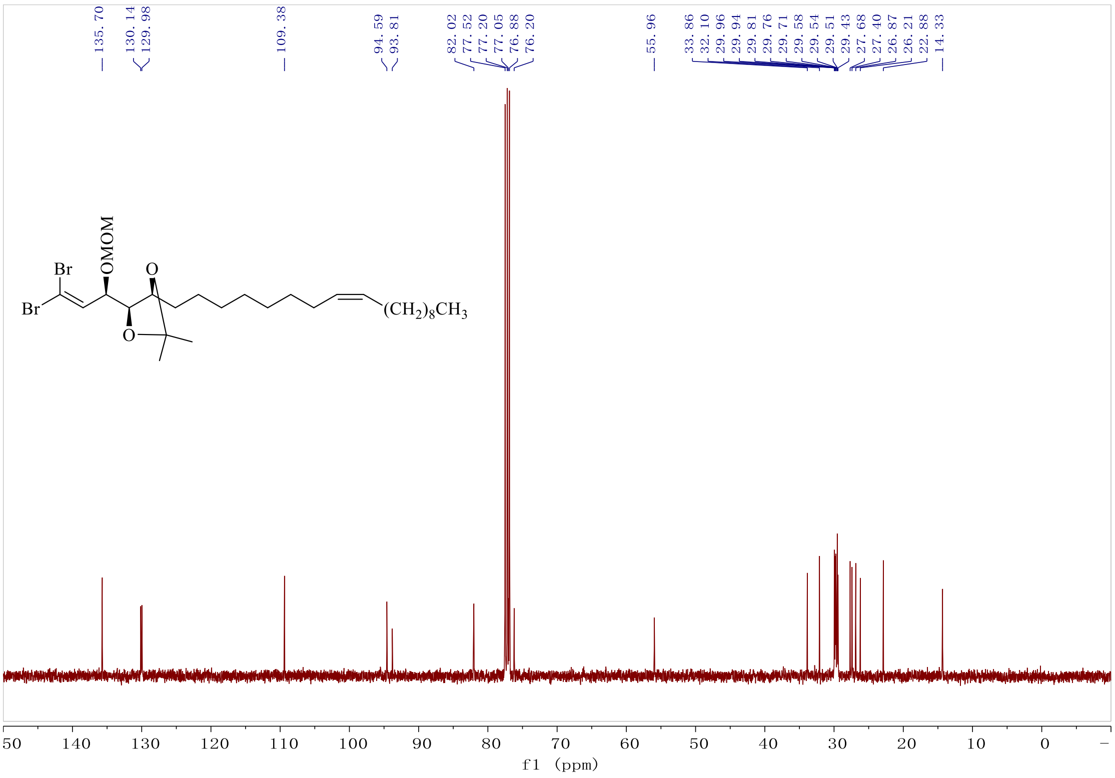

^{1}H and ^{13}C NMR ($CDCl_3$) Spectra of Compound **17a**

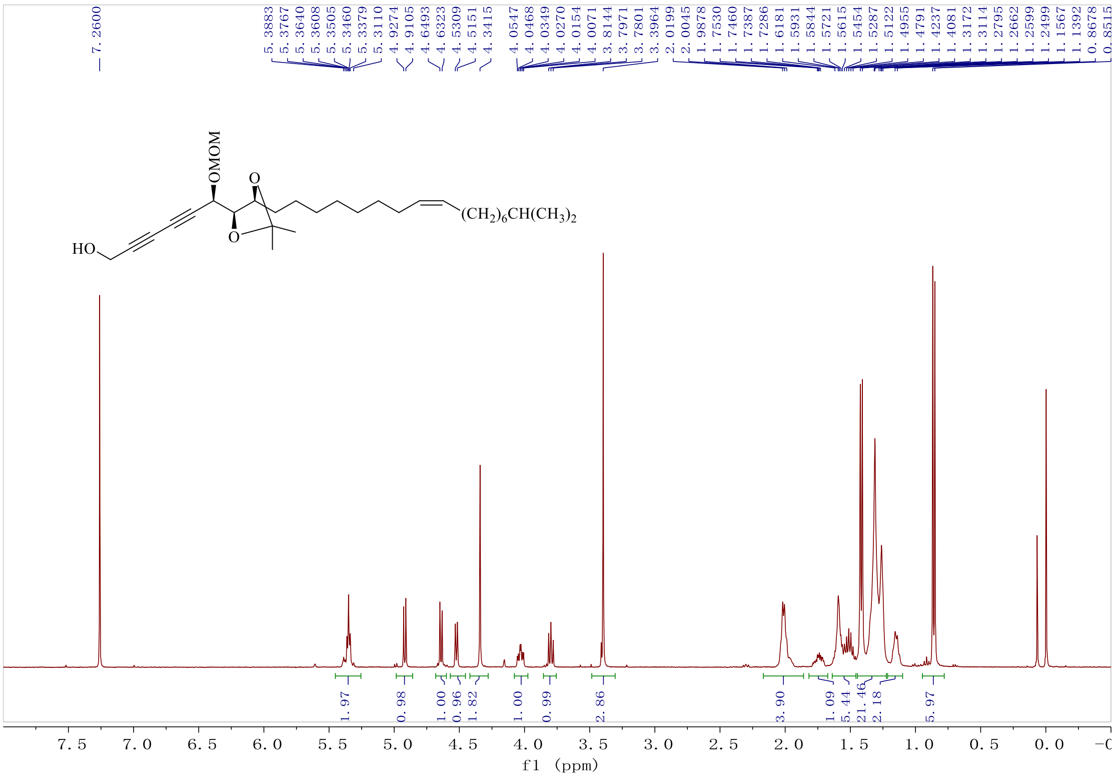

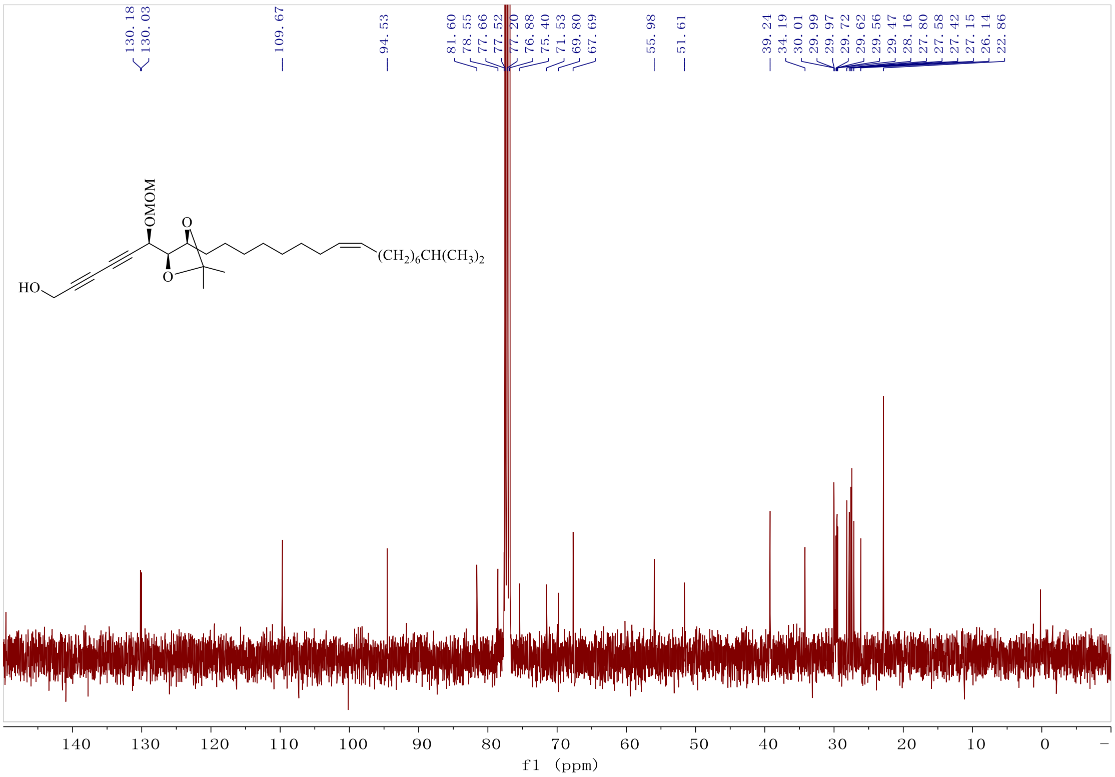

^{1}H and ^{13}C NMR ($CDCl_3$) Spectra of Compound **17b**

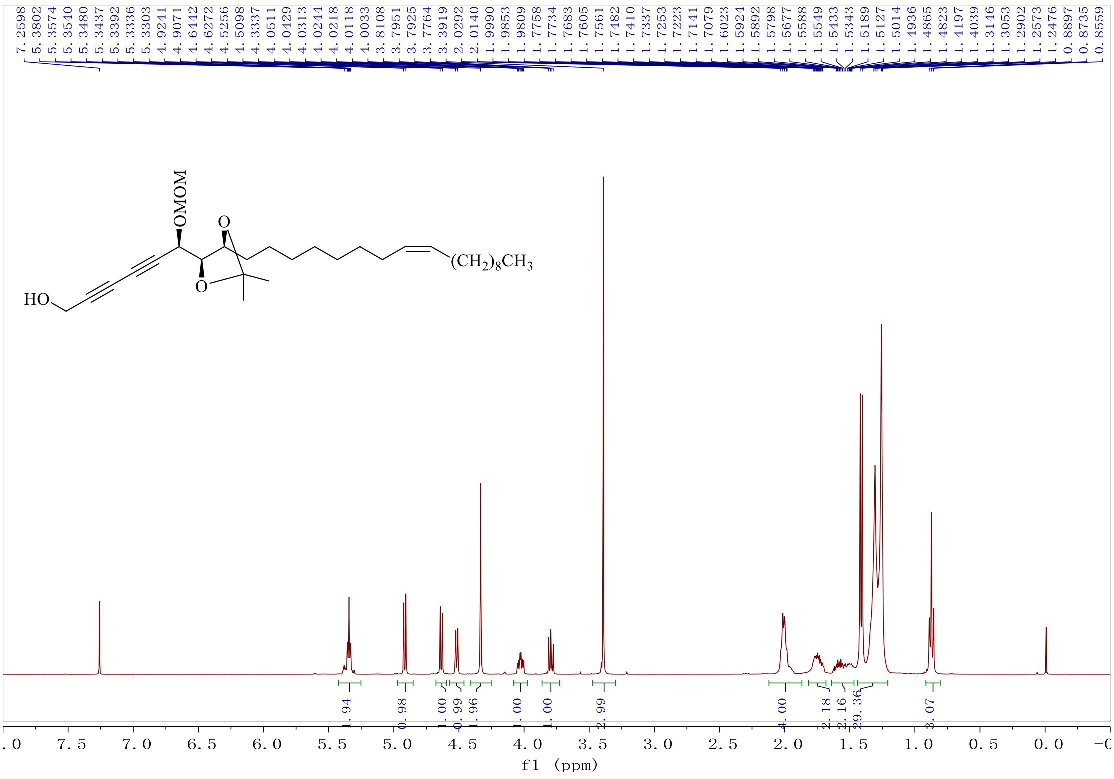

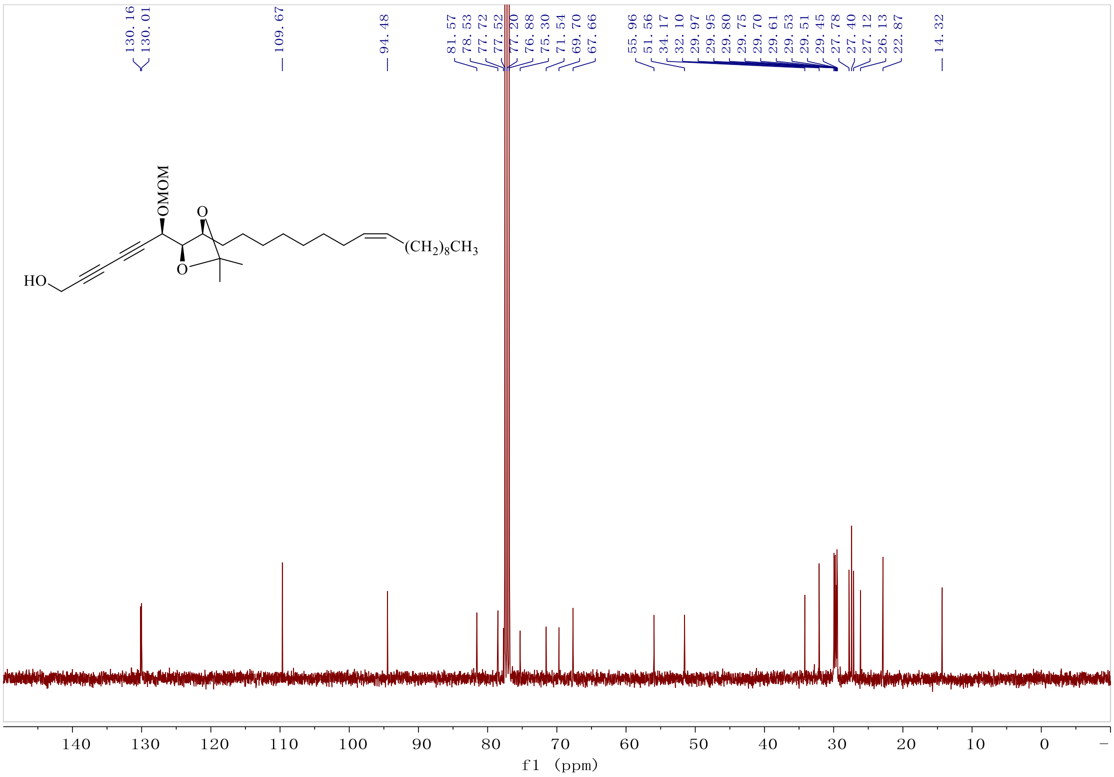

^{1}H and ^{13}C NMR ($CDCl_3$/ MeOD =4:1) Spectra of Compound **2**

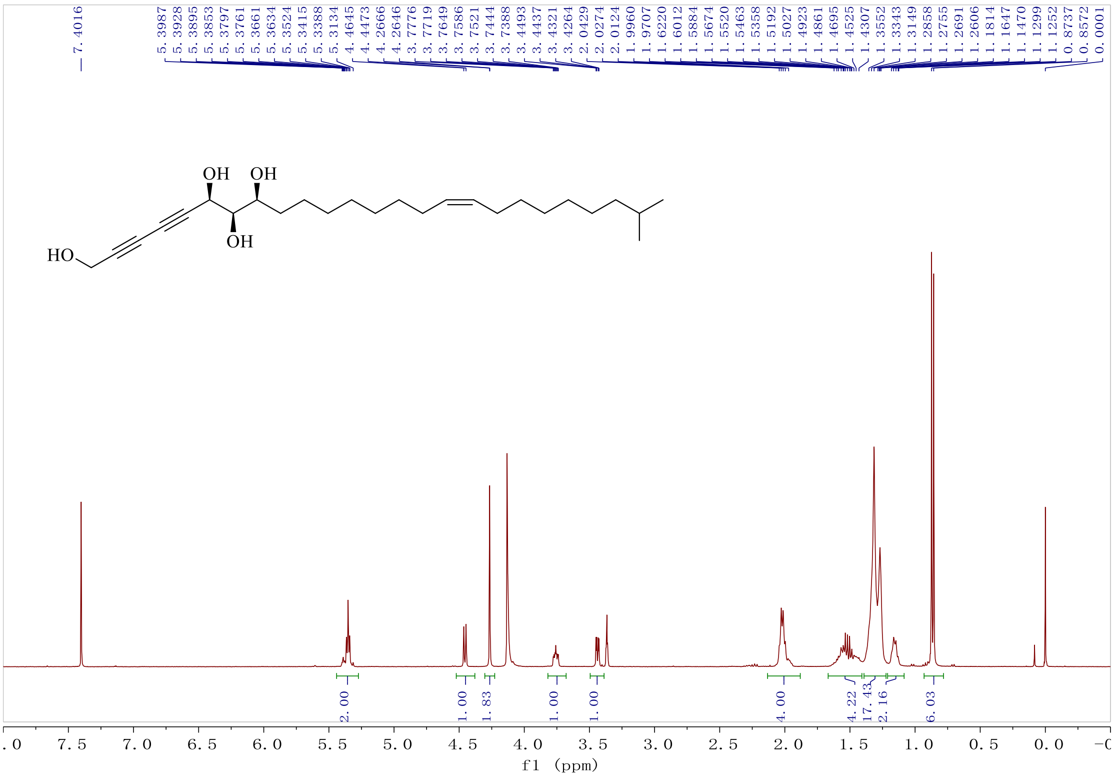

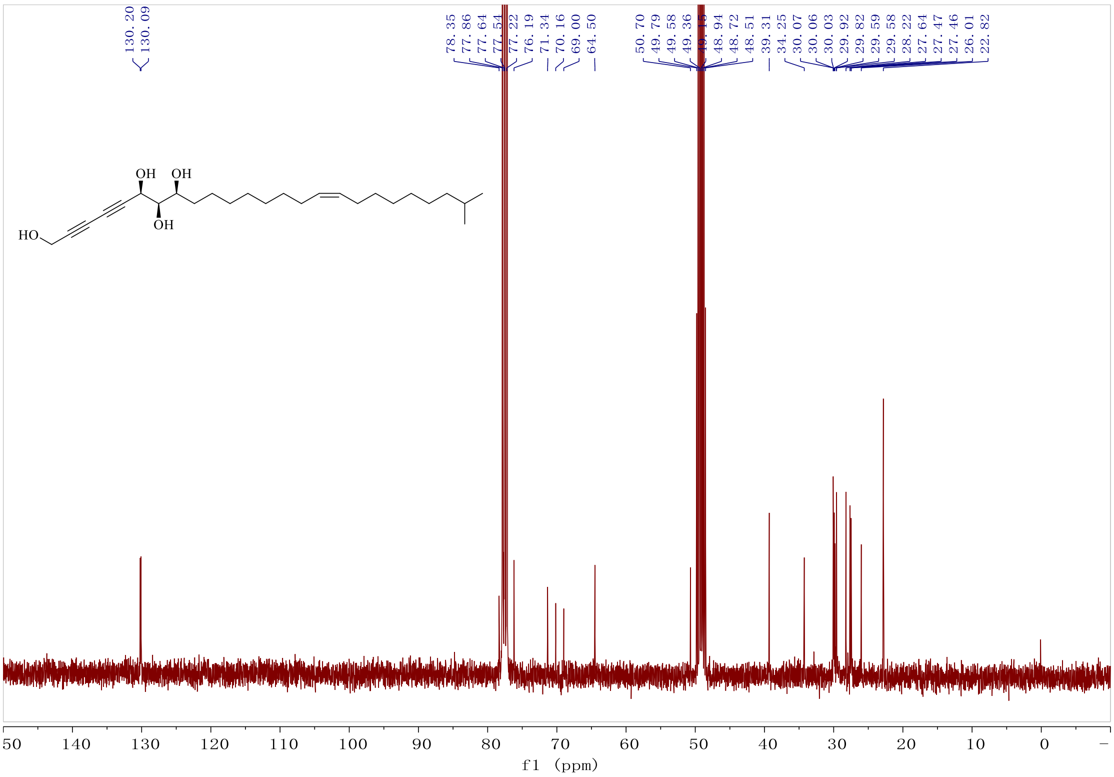

^{1}H and ^{13}C NMR (MeOD) Spectra of Compound **2**

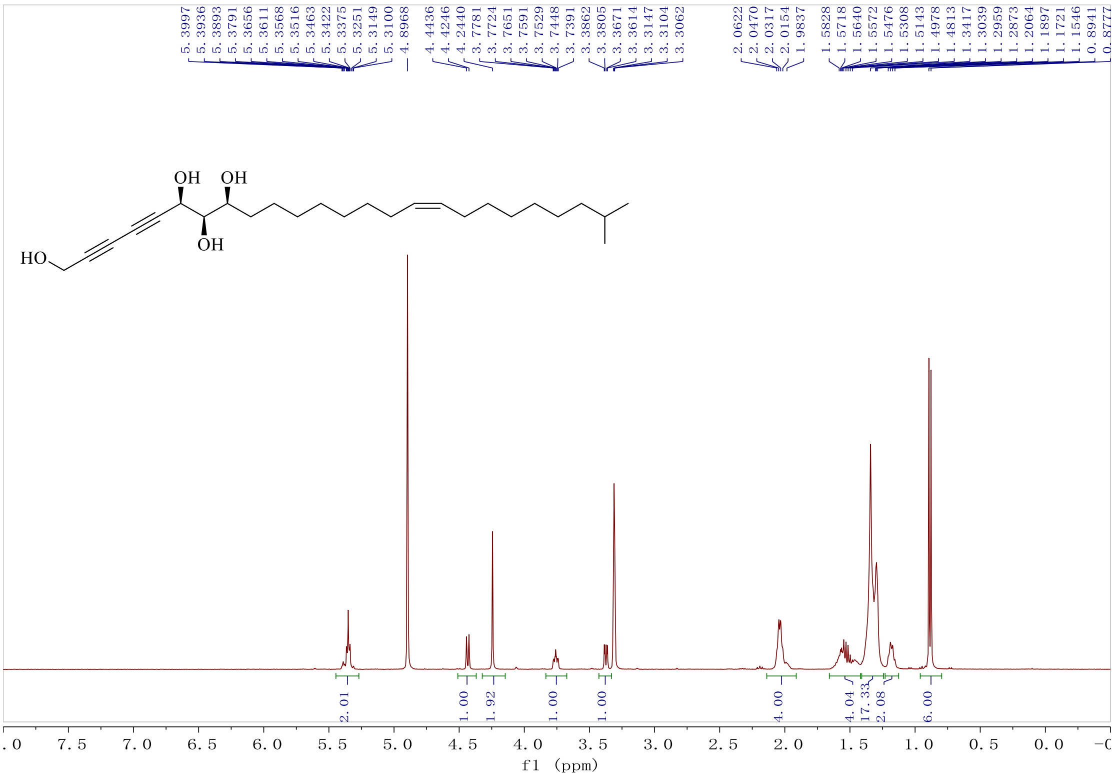

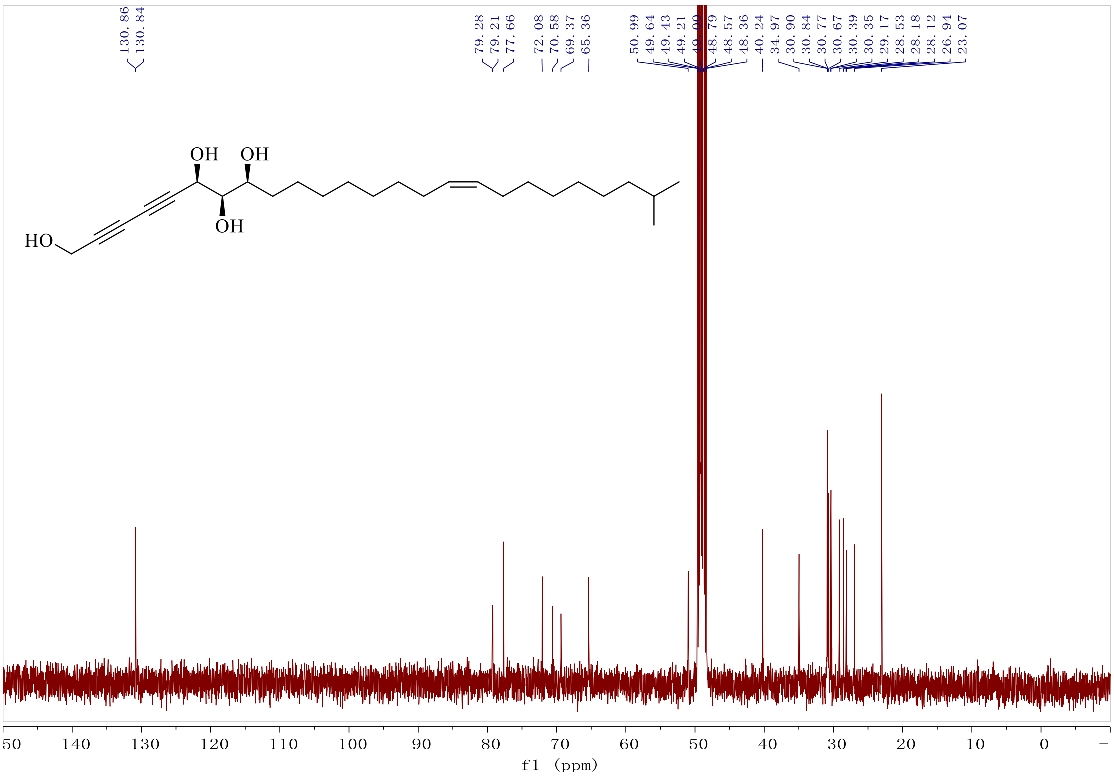

^{1}H and ^{13}C NMR ($CDCl_3$/ MeOD =4:1) Spectra of Compound **4**

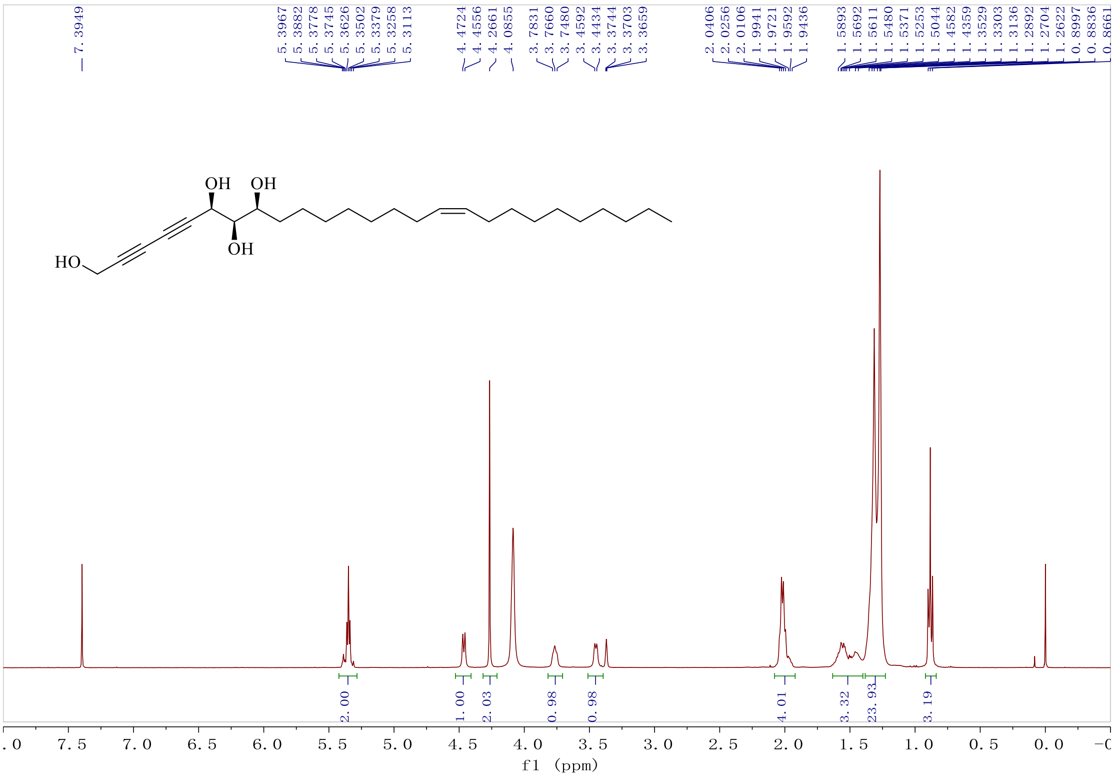

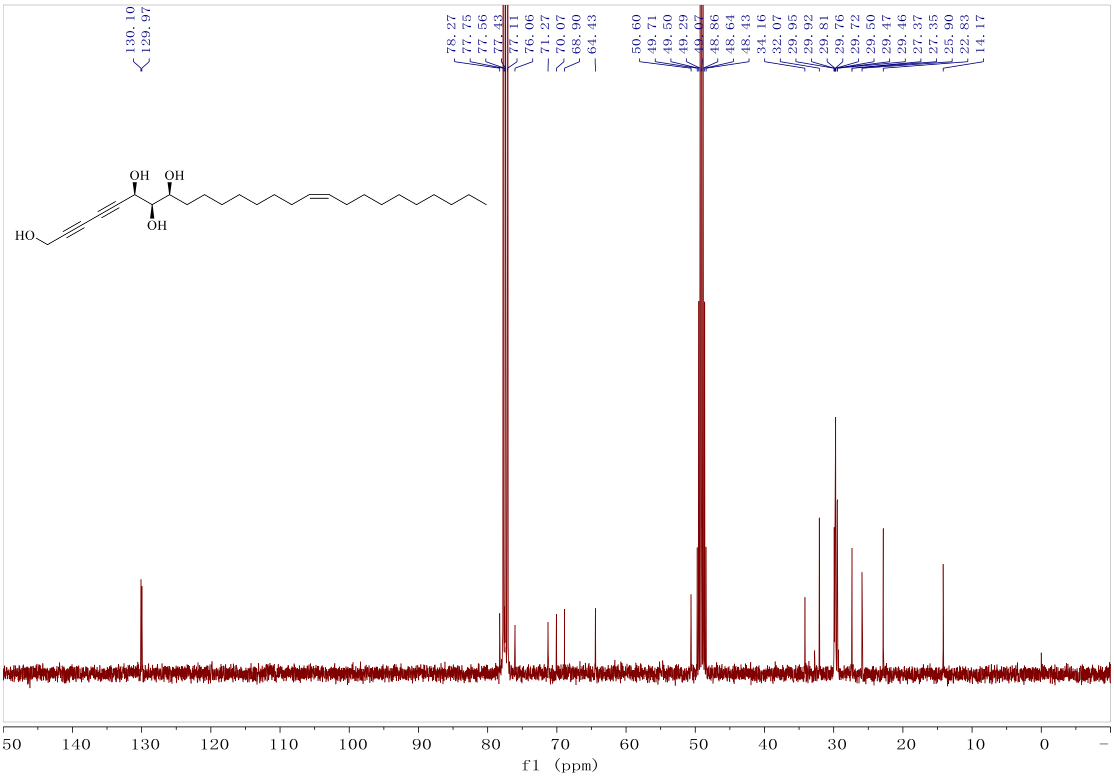

^{1}H and ^{13}C NMR (MeOD) Spectra of Compound **4**

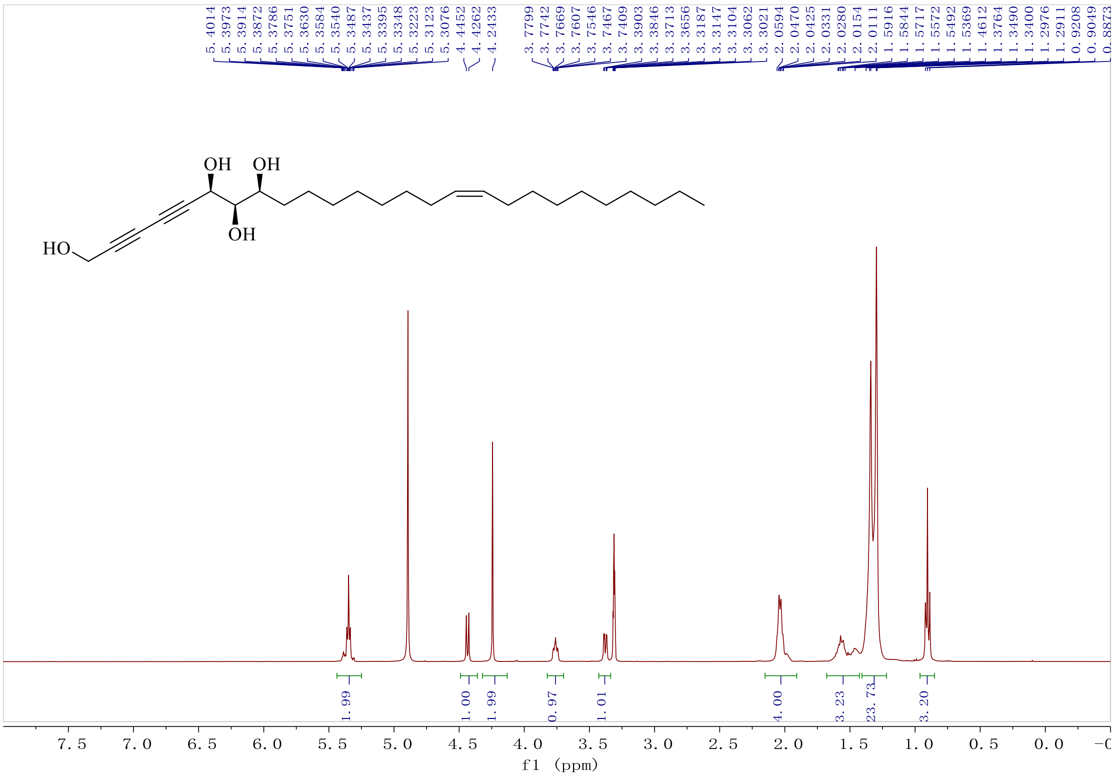

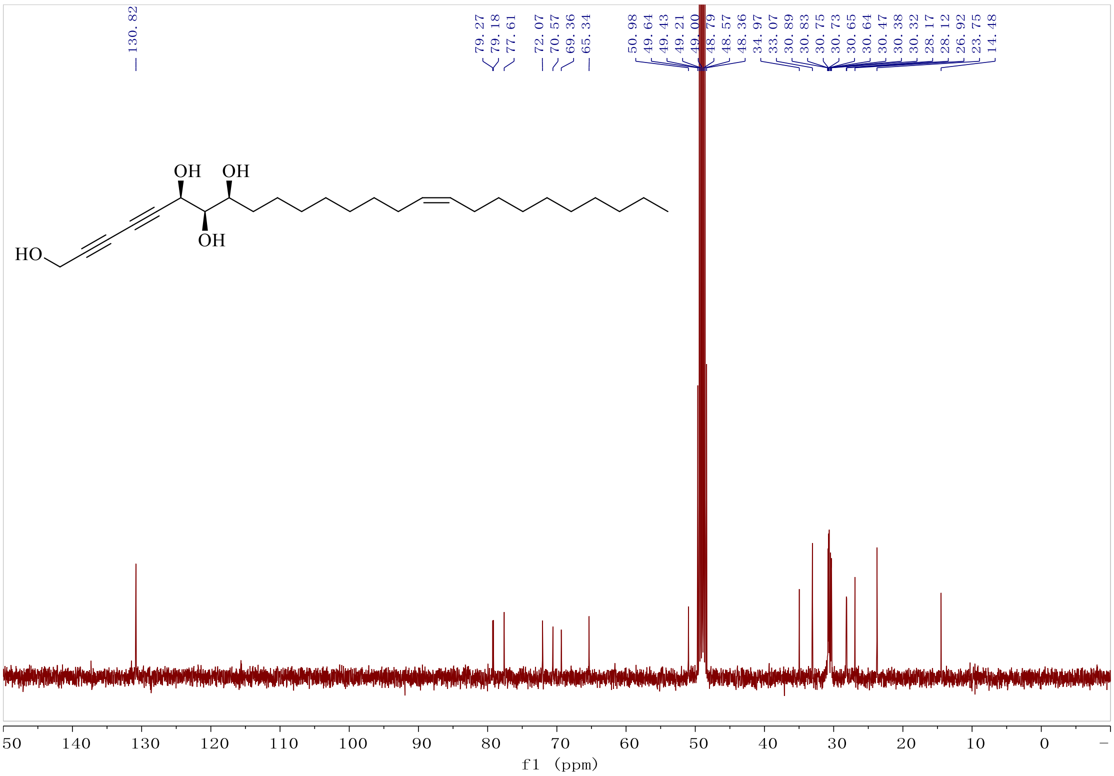

Comparison of ^{1}H and ^{13}C NMR data of natural and synthetic petrosiol B (**2**)

Table 1. Comparison of ^{1}H and ^{13}C NMR data of natural[a] and synthetic[b] petrosiol B (**2**).

No	^{1}H-natural	^{1}H-synthetic	^{13}C-natural	^{13}C-synthetic
1	4.26(s)	4.27(s)	50.7	50.7
2	-		78.4	78.4
3	-		69.0	69.0
4	-		70.1	70.2
5	-		77.5	77.6
6	4.46(d,6.6)	4.46(d,6.9)	64.5	64.5
7	3.44(dd,6.6,1.8)	3.44(dd,6.9,2.2)	76.2	76.2
8	3.76(m)	3.75(m)	71.3	71.3
9	1.57(m)	1.55 (m)	34.2	34.3
10	1.34(m)	1.2-1.4(m)		
	1.46(m)	1.2-1.4(m)	25.9	26.0
11-14	1.2-1.4(m)	1.2-1.4(m)	29.5-30.2	29.6-30.1
15	2.02(m)	2.01(m)	27.4	27.5
16	5.35(m)	5.36(m)	130.0	130.1
17	5.35(m)	5.36(m)	130.2	130.2
18	2.02(m)	2.01(m)	27.4	27.5
19	1.2-1.4(m)	1.2-1.4(m)	29.5-30.0	29.6-30.1
20	1.2-1.4(m)	1.2-1.4(m)	29.5-30.0	29.6-30.1
21	1.2-1.4(m)	1.2-1.4(m)	29.5-30.0	29.6-30.1
22	1.2-1.4(m)	1.2-1.4(m)	27.6	27.6
23	1.16(m)	1.15(m)	39.3	39.3
24	1.52(m)	1.55(m)	28.2	28.2
25	0.87(d,6.6)	0.87(d,6.6)	22.7	22.8
26	0.87(d,6.6)	0.87(d,6.6)	22.7	22.8

[a]NMR data were recorded at 600 MHz (^{1}H NMR) and 150 MHz (^{13}C NMR) in $CDCl_3$/MeOD (4:1).

[b]NMR data were recorded at 400 MHz (^{1}H NMR) and 100 MHz (^{13}C NMR) in $CDCl_3$/MeOD (4:1).

Comparison of ^{1}H and ^{13}C NMR data of natural and synthetic petrosiol D (**4**)

Table 2. Comparison of ^{1}H and ^{13}C NMR data of natural[a] and synthetic[b] petrosiol D (**4**).

No	^{1}H-natural	^{1}H-synthetic	^{13}C-natural	^{13}C-synthetic
1	4.27(s)	4.27(s)	50.7	50.6
2	-		78.4	78.3
3	-		69.0	68.9
4	-		70.1	70.1
5	-		77.6	77.6
6	4.46(d,6.6)	4.46(d,6.7)	64.5	64.4
7	3.44(dd,6.6,1.8)	3.45(d,6.3)	76.2	76.1
8	3.76(m)	3.77(m)	71.3	71.3
9	1.55(m)	1.7-1.4 (m)	34.2	34.2
10	1.34(m)	1.2-1.4(m)		
	1.46(m)	1.7-1.4 (m)	25.9	25.9
11-14	1.2-1.4(m)	1.2-1.4(m)	29.5-30.2	29.5-30.0
15	2.02(m)	2.00(m)	27.4	27.4
16	5.35(m)	5.36(m)	130.0	130.0
17	5.35(m)	5.36(m)	130.2	130.1
18	2.02(m)	2.00(m)	27.4	27.4
19	1.2-1.4(m)	1.2-1.4(m)	29.5-30.2	29.5-30.0
20	1.2-1.4(m)	1.2-1.4(m)	29.5-30.2	29.5-30.0
21	1.2-1.4(m)	1.2-1.4(m)	29.5-30.2	29.5-30.0
22	1.2-1.4(m)	1.2-1.4(m)	29.5-30.2	29.5-30.0
23	1.2-1.4 (m)	1.2-1.4(m)	29.5-30.2	29.5-30.0
24	1.2-1.4(m)	1.2-1.4(m)	32.1	32.1
25	1.2-1.4(m)	1.2-1.4(m)	22.9	22.8
26	0.89(t,6.6)	0.88(t,6.7)	14.2	14.2

[a]NMR data were recorded at 600 MHz (^{1}H NMR) and 150 MHz (^{13}C NMR) in $CDCl_3$/MeOD (4:1).

[b]NMR data were recorded at 400 MHz (^{1}H NMR) and 100 MHz (^{13}C NMR) in $CDCl_3$/MeOD (4:1).
